# Diagnostic Accuracy of Exercise Stress Testing, Stress Echocardiography, Myocardial Scintigraphy, and Cardiac Magnetic Resonance for Obstructive Coronary Artery Disease: Systematic Reviews and Meta-Analyses of 104 Studies Published from 1990 to 2025

**DOI:** 10.3390/jcm14176238

**Published:** 2025-09-04

**Authors:** Andrea Sonaglioni, Alessio Polymeropoulos, Massimo Baravelli, Gian Luigi Nicolosi, Michele Lombardo, Giuseppe Biondi-Zoccai

**Affiliations:** 1 Division of Cardiology, IRCCS MultiMedica, 20123 Milan, Italy; massimo.baravelli@multimedica.it (M.B.); michele.lombardo@multimedica.it (M.L.); 2 Department of Statistics and Quantitative Methods, University of Milano-Bicocca, 20126 Milan, Italy; a.polymeropoulos@campus.unimib.it; 3 Division of Cardiology, Policlinico San Giorgio, 33170 Pordenone, Italy; gianluigi.nicolosi@gmail.com; 4 Department of Medico-Surgical Sciences and Biotechnologies, Sapienza University, 04100 Latina, Italy; giuseppe.biondizoccai@uniroma1.it; 5 Maria Cecilia Hospital, GVM Care & Research, 48033 Cotignola, Italy

**Keywords:** coronary artery disease, diagnostic accuracy, exercise stress testing, stress echocardiography, myocardial SPECT, stress CMR, meta-analysis

## Abstract

**Background:** Since the 1990s, numerous investigations have assessed the diagnostic effectiveness—specifically sensitivity, specificity, and accuracy—of exercise stress testing (EST), stress echocardiography (SE), stress myocardial single-photon emission computed tomography (SPECT), and stress cardiac magnetic resonance imaging (CMR). However, the outcomes of these studies have often been inconsistent and inconclusive. To provide a clearer comparison, we conducted systematic reviews and meta-analyses aimed at quantitatively evaluating and comparing the aggregated diagnostic performance of these four commonly used techniques for detecting coronary artery disease (CAD). **Methods:** A comprehensive search of PubMed, Scopus, Embase, Cochrane Library, and Web of Science was conducted to identify cohort studies evaluating the diagnostic accuracy of EST, SE, stress myocardial SPECT, and stress CMR in symptomatic patients with suspected or confirmed CAD. The main goal was to compare their diagnostic value by pooling sensitivity and specificity results. Each study’s data were extracted in terms of true positives, false positives, true negatives, and false negatives. **Results:** A total of 104 studies, comprising 16,824 symptomatic individuals with either suspected or known CAD, met the inclusion criteria. The pooled sensitivities for CAD detection were 0.66 (95% CI: 0.59–0.72, *p* < 0.001) for EST, 0.81 (95% CI: 0.79–0.83, *p* < 0.001) for SE, 0.82 (95% CI: 0.78–0.85, *p* < 0.001) for stress myocardial SPECT, and 0.83 (95% CI: 0.81–0.85, *p* < 0.001) for stress CMR. Corresponding specificities were 0.61 (95% CI: 0.55–0.67, *p* < 0.001), 0.85 (95% CI: 0.82–0.87, *p* < 0.001), 0.74 (95% CI: 0.70–0.78, *p* < 0.001), and 0.89 (95% CI: 0.86–0.92, *p* < 0.001), respectively. Considerable heterogeneity was observed across the studies, as reflected by I^2^ values ranging from 82.5% to 92.5%. Egger’s generalized test revealed statistically significant publication bias (*p* < 0.05 for all methods), likely due to the influence of smaller studies reporting more favorable results. Despite this, sensitivity analyses supported the overall robustness and reliability of the pooled findings. **Conclusions:** Among the diagnostic tools assessed, EST demonstrated the lowest accuracy for detecting obstructive CAD, whereas stress CMR exhibited the highest. Although stress myocardial SPECT showed strong sensitivity, its specificity was comparatively limited. SE emerged as the most balanced option, offering good diagnostic accuracy combined with advantages such as broad availability, cost-effectiveness, and the absence of ionizing radiation.

## 1. Introduction

Coronary artery disease (CAD) remains the leading cause of death globally [1], affecting an estimated 200 million individuals worldwide [2]. Prevalence rates vary by region, with Central and Eastern Europe and Central Asia experiencing the highest burden, while South Asia reports the lowest [3]. The global incidence of CAD is projected to increase through 2025–2034, driven by aging populations and ongoing exposure to risk factors [4]. Early screening plays a vital role in minimizing both morbidity and mortality by enabling timely interventions, such as lifestyle modifications, cardioprotective therapies, and coronary revascularization procedures. Assessing the pre-test probability of CAD—based on variables like age, gender, symptoms, and cardiovascular risk profile—can help guide clinicians toward the most suitable diagnostic approach [5].

According to the 2024 European Guidelines [6], diagnostic testing is advised for symptomatic patients with a moderate (15–50%) or high (50–85%) likelihood of obstructive CAD, while those with a low likelihood (5–15%) may not require immediate testing unless symptoms persist or are unclear. The guidelines particularly endorse coronary computed tomography angiography (CCTA) for symptomatic individuals within the low to moderate (5–50%) pre-test likelihood range. For individuals assessed to have a very low likelihood (≤5%) of obstructive CAD, screening may be postponed unless symptoms continue and non-cardiac origins have been excluded.

Despite these evidence-based recommendations, a substantial number of functional diagnostic tests are still conducted for reasons deemed “rarely appropriate” [7,8,9,10]. This trend may reflect limited adherence to guidelines by healthcare professionals, the comparatively lower availability of CCTA versus conventional functional tests and the influence of defensive medicine, which can lead to unnecessary procedures and inflated healthcare costs [11,12]. Currently, the most widely used noninvasive functional tests for detecting obstructive CAD include exercise stress testing (EST), stress echocardiography (SE), stress myocardial perfusion imaging with single-photon emission computed tomography (SPECT), and stress cardiac magnetic resonance (CMR). These tests employ stress agents—such as physical exercise, dobutamine, dipyridamole, or adenosine—to provoke myocardial ischemia in symptomatic patients.

Since the 1990s, numerous studies have examined the diagnostic performance—sensitivity, specificity, and accuracy—of EST, SE, stress myocardial SPECT, and stress CMR, with results often inconsistent and inconclusive. To address this, we propose conducting systematic reviews and meta-analyses to quantitatively compare the pooled diagnostic metrics of each method used for CAD detection. Furthermore, we will explore the technical and physiological mechanisms underlying the differences in diagnostic accuracy among the four primary testing modalities and their associated ischemic stressors.

## 2. Materials and Methods

These systematic reviews and meta-analyses adhered to the Preferred Reporting Items for Systematic Reviews and Meta-Analyses (PRISMA) guidelines [13] and were officially registered with the PROSPERO database (registration number: CRD420251118642).

### 2.1. Search Strategy

Two independent reviewers (A.S. and M.B.) conducted comprehensive literature searches across PubMed, Scopus, Embase, Cochrane, and Web of Science. They reviewed all available studies—without restrictions on publication date—that evaluated the diagnostic sensitivity, specificity, and overall accuracy of four main noninvasive tests: EST, SE, myocardial SPECT, and stress CMR. The analyses also focused on four primary ischemic stress agents: physical exercise, dobutamine, dipyridamole, and adenosine. The search, performed between 3 June and 27 June 2025, incorporated terms such as “sensitivity”, “specificity”, “accuracy”, “coronary artery disease”, or “CAD”, and related keywords for each imaging modality and stress agent. No language filters were applied, and pediatric studies were also considered eligible.

### 2.2. Inclusion and Exclusion Criteria

Eligible studies were those evaluating the diagnostic accuracy of the four primary tests (EST, SE, stress myocardial SPECT, and stress CMR) in symptomatic individuals with suspected or known CAD. Excluded were studies lacking complete diagnostic data, those without coronary angiography confirmation, animal research, non-original articles, duplicate records, abstracts, case reports, conference materials, reviews, editorials, and letters without primary data.

### 2.3. Study Selection and Data Extraction

Study selection and data extraction were conducted independently by A.S. and M.B. Extracted data included the following: (1) patient demographics (age and sex); (2) history of CAD (e.g., previous myocardial infarction or revascularization); (3) the threshold used for defining obstructive CAD (≥50% or ≥70% stenosis); (4) sensitivity = true positives (TP)/[TP + false negatives (FN)]; (5) specificity = true negatives (TN)/[TN + false positives (FP)]; (6) diagnostic accuracy = (TP + TN)/total sample; and (7) follow-up details, if available. Any discrepancies were resolved through consultation with a third reviewer (G.L.N.), who also verified all extracted information.

### 2.4. Assessment of Bias

The risk of bias for included studies was assessed using the National Institutes of Health (NIH) Quality Assessment Tool for Observational Cohort and Cross-Sectional Studies [14]. A.S. and G.L.N. independently rated each study against 14 criteria, classifying overall quality as “good” (11–14 criteria met), “fair” (6–10), or “poor” (0–5). Discrepancies were resolved through discussion. Inter-rater reliability was assessed using Cohen’s Kappa coefficient (k), calculated with the formula k = (p_o_ − p_e_)/(1 − p_e_), where p_o_ is the observed agreement and p_e_ is the expected agreement by chance [15].

### 2.5. Statistical Methods

The primary objective was to assess the diagnostic performance of the four commonly used tests (EST, SE, stress myocardial SPECT, and stress CMR) by analyzing pooled sensitivity and specificity data [16,17]. TP, TN, FP, and FN were extracted as raw counts for each included study.

A Bayesian bivariate meta-analysis using a binomial–normal framework was applied to jointly model sensitivity and specificity, building on prior research [18,19]. The model used a logit link function with non-informative normal priors (with a mean of zero and large variance) for fixed effects and penalized complexity priors (hyperparameters 3 and 0.05) for random-effect variances. For the Fisher’s z-transformed correlation, a normal prior with a zero mean and large variance was assumed.

Diagnostic performance was visually summarized using forest plots [20], crosshairs plots [21] and summary receiver operating characteristic (SROC) curves [22]. Crosshairs plots were used to visualize study-level sensitivity and specificity estimates, while forest plots presented pooled values graphically. The area under each SROC curve (AUC) was calculated to compare the overall diagnostic effectiveness of different tests and stress agents.

For each meta-analysis of methods, model fitting was refined using 40,000 iterations of the Markov Chain Monte Carlo (MCMC) procedure.

Posterior estimates for sensitivity and specificity were calculated for all four modalities. These estimates were also stratified in subgroup analyses to explore sources of heterogeneity. For each modality, the following subgroups were assessed: (1) EST: studies involving both sexes vs. female-only samples; (2) SE: based on type of stress (exercise, dobutamine, dipyridamole, or dual imaging); (3) SPECT: stratified by stressor (exercise, dobutamine, dipyridamole, or adenosine); and (4) CMR: stratified similarly to SPECT.

The I^2^ statistic was used to assess the degree of variability among studies in the subgroup analysis, attributing it to heterogeneity rather than random chance, since Bayesian approaches do not offer a sufficient measure of heterogeneity.

Bivariate funnel plots [23] were used to assess the risk of publication bias based on study precision. Egger’s generalized test, suitable for bivariate structures, was employed to statistically evaluate publication bias (https://doi.org/10.48550/arXiv.2209.07270) [24].

To evaluate robustness, sensitivity analyses were performed by iteratively excluding individual studies and re-running the meta-analysis [25]. Posterior credibility intervals and confidence intervals were computed, and a two-sided *p* value < 0.05 was considered statistically significant where applicable.

All statistical analyses were conducted using Comprehensive Meta-Analysis software version 3.0 (Biostat, Englewood, NJ, USA) and the *R* programming language (v4.3.2), employing the meta4diag package (v2.1.1) for diagnostic test meta-analysis.

## 3. Results

### 3.1. Study Selection

**Figure 1:**
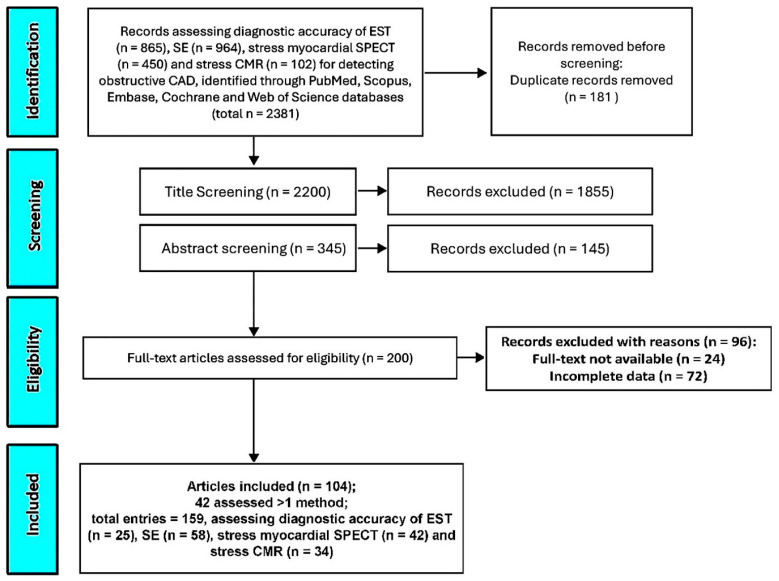
The PRISMA flow diagram used for identifying the included studies. CAD, coronary artery disease; CMR, cardiac magnetic resonance; EST, exercise stress testing; SE, stress echocardiography; and SPECT, single-photon emission computed tomography.

A comprehensive search was conducted across PubMed, Scopus, Embase, Cochrane, and Web of Science databases to identify studies evaluating the sensitivity, specificity, and diagnostic accuracy of EST, SE, stress myocardial SPECT, and stress CMR in detecting obstructive CAD. This initial search yielded 2381 records. After removing 181 duplicate entries (7.6%), an additional 1855 studies (77.9%) were excluded based on title screening, and 145 (6.1%) were removed following abstract review. The remaining 200 studies (8.4%) underwent full-text evaluation for eligibility. Of these, 24 (1%) were excluded due to unavailable full texts and 72 (3%) due to incomplete or missing data. Ultimately, 104 studies (4.4%) [26,27,28,29,30,31,32,33,34,35,36,37,38,39,40,41,42,43,44,45,46,47,48,49,50,51,52,53,54,55,56,57,58,59,60,61,62,63,64,65,66,67,68,69,70,71,72,73,74,75,76,77,78,79,80,81,82,83,84,85,86,87,88,89,90,91,92,93,94,95,96,97,98,99,100,101,102,103,104,105,106,107,108,109,110,111,112,113,114,115,116,117,118,119,120,121,122,123,124,125,126,127,128,129] involving a total of 16,824 symptomatic patients with suspected or known CAD were included in the systematic review and meta-analysis. Notably, 42 studies (40.4%) examined more than one diagnostic modality, resulting in 159 total analyses evaluating the performance of EST (n = 25), SE (n = 58), stress myocardial SPECT (n = 42), and stress CMR (n = 34) (Figure 1).

### 3.2. Clinical Characteristics, Stress Protocols, and Main Findings of the Included Studies

Table 1 provides an overview of the included studies, outlining key information such as the study name, year of publication, country of origin, study design, sample size, percentage of female participants, average patient age, and whether CAD was suspected or previously diagnosed. It also details the angiographic criteria used to define obstructive CAD, the stress testing protocols applied, and the reported sensitivity, specificity, and accuracy for each myocardial ischemia detection method. For the 42 studies that assessed more than one diagnostic technique, the findings are presented separately for each method examined.

The 104 studies included in the analysis were published between 1990 and 2025. Among these, 27 were conducted in the United States, 16 in Italy, 12 in Germany, 7 each in The Netherlands and the United Kingdom, 4 each in Switzerland and Spain, and 3 in Turkey, Greece, and Japan. Two studies originated from Belgium, Serbia, Poland, China, and Taiwan, while single studies were reported from France, Ireland, Norway, Portugal, Argentina, Chile, Canada, and Iran. The average patient age across all studies was 59.4 years (ranging from 14.1 to 71 years), with women comprising 33.9% of the study population (range: 1.9% to 100%).

Most of the included studies (91.4%) were prospective in design, with only nine (8.6%) being retrospective. Furthermore, 90 studies (86.5%) were single-center investigations, while 14 (13.5%) involved multiple centers. Roughly half of the studies (51.9%) enrolled symptomatic patients without a prior history of CAD, while 6.7% included only individuals with established CAD. The remaining 41.4% evaluated symptomatic patients with either known or suspected CAD. In terms of coronary angiographic criteria, the majority of studies (72.1%) defined significant coronary stenosis as a ≥50% reduction in luminal diameter. Two studies (1.9%) used a ≥60% threshold, 23 studies (22.1%) applied a ≥70% cutoff, and four studies (3.8%) used a ≥75% definition for obstructive CAD.

The stress testing protocols employed varied across modalities. For EST, exercise SPECT, and exercise CMR, the standard Bruce protocol was most frequently used, incorporating 25-Watt incremental workloads every 2 min. Supine bicycle ergometry with the same 25-Watt incremental approach on a tilting table was commonly used in exercise stress echocardiography (ESE). Dobutamine stress testing protocols typically involved an IV infusion starting at 5 mcg/kg/min and increasing to 40 mcg/kg/min in 3 min stages, with the addition of atropine up to 1 mg for dobutamine echo and SPECT studies, and up to 2 mg for CMR protocols. Dipyridamole-based protocols included a two-step IV infusion ranging from 0.56 to 0.84 mg/kg over 10 min plus atropine (0.25–1 mg) for dipyridamole echo and SPECT, while a single-step 0.56 mg/kg over 4 min was applied for dipyridamole CMR. Adenosine was administered at 140 mcg/kg/min over 4–6 min in both SPECT and CMR stress testing protocols.

Among all assessed modalities, EST demonstrated the lowest overall diagnostic performance, with a pooled sensitivity, specificity, and accuracy of 63.6%, 62.3%, and 63.2%, respectively, for detecting obstructive CAD. In contrast, dipyridamole stress CMR showed the highest pooled sensitivity (84.8%) and diagnostic accuracy (87.3%). Notably, exercise CMR yielded the highest pooled specificity (92.2%) across all tests in identifying obstructive CAD.

### 3.3. NIH Quality Rating

Based on the NIH quality assessment tool, 73 studies were rated as having good methodological quality, while the remaining 31 were classified as fair (see Supplementary Table S1). The level of inter-reviewer agreement for the risk of bias evaluation was substantial, as reflected by a Cohen’s Kappa coefficient of 0.82.

### 3.4. Diagnostic Accuracy of Exercise Stress Testing for Detecting Obstructive CAD

**Figure 2:**
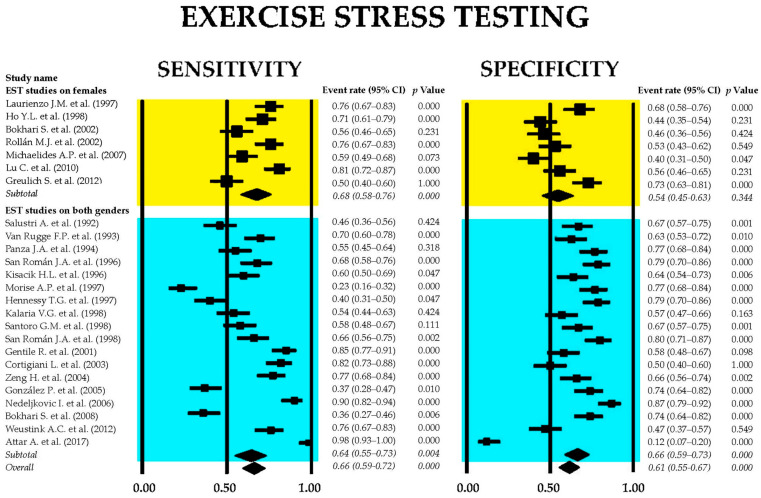
Forest plots illustrating the sensitivity and specificity of exercise stress testing are provided for the overall set of studies [26,27,28,29,30,31,32,33,34,35,36,37,38,39,40,41,42,43,44,45,46,47,48,49,50], as well as for the subgroups comprising female-only participants [31,34,39,40,45,47,48] and mixed-gender [26,27,28,29,30,32,33,35,36,37,38,41,42,43,44,45,46,49,50] cohorts.

Figure 2 presents the forest plots depicting the sensitivity and specificity of EST across all included studies, as well as within the two subgroups: studies involving only female participants and those including both sexes.

The combined sensitivity of EST for identifying obstructive CAD was 0.66 (95% CI: 0.59–0.72, *p* < 0.001), while the pooled specificity was 0.61 (95% CI: 0.55–0.67, *p* < 0.001). Subgroup analysis indicated that studies focused exclusively on female participants demonstrated low specificity, which did not reach statistical significance. The heterogeneity across studies was substantial, with I^2^ values of 91.5% for sensitivity and 89.5% for specificity (both *p* < 0.001).

**Figure 3:**
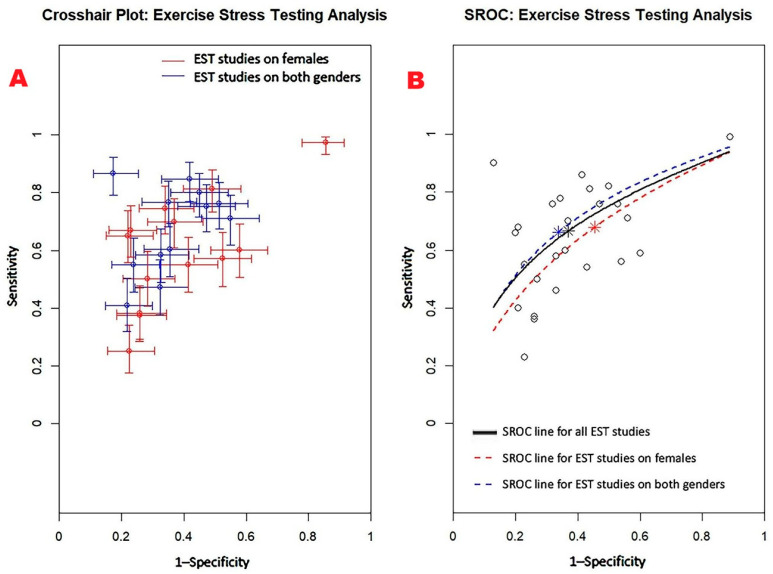
(**A**) Crosshair plot illustrating the results of EST studies conducted in two distinct subgroups—female-only and mixed-gender populations. (**B**) The SROC curve represents the overall diagnostic performance of EST across all studies (AUC = 0.69; 95% CI: 0.62–0.76), as well as for studies including only women (AUC = 0.65; 95% CI: 0.52–0.80) and those involving both sexes (AUC = 0.71; 95% CI: 0.61–0.78). EST, exercise stress testing; SROC, summary receiver operating characteristic.

Figure 3 displays the crosshair plot (A), illustrating the performance of EST in the subgroups of female-only and mixed-gender populations, along with the SROC curve (B), which consolidates the diagnostic performance data from all included EST studies as well as from the respective subgroups.

**Figure 4:**
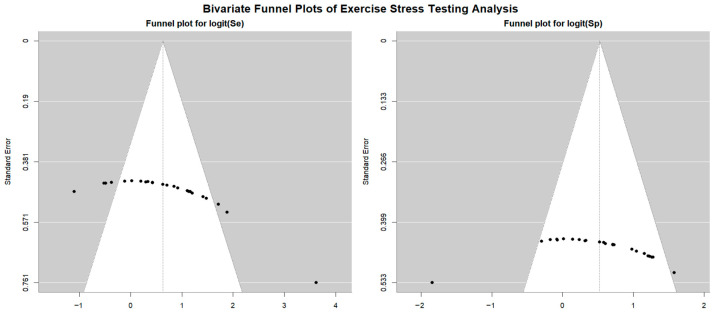
Begg’s funnel plot for the detection of publication bias in EST studies.

Figure 4 shows Begg’s funnel plot for the detection of publication bias in EST studies.

Egger’s generalized test indicated potential publication bias with a *p* value of 0.02. A sensitivity analysis supported the stability of the study findings. The exclusion of individual studies led to only minor fluctuations in diagnostic estimates. Specifically, in EST studies involving female participants, sensitivity ranged from 0.68 (95% CI: 0.55–0.78, *p* = 0.007) to 0.73 (95% CI: 0.67–0.79, *p* < 0.001), while specificity varied from 0.50 (95% CI: 0.41–0.60, *p* = 0.96) to 0.56 (95% CI: 0.33–0.77, *p* = 0.61). In studies that included both sexes, sensitivity ranged from 0.52 (95% CI: 0.39–0.64, *p* = 0.80) to 0.62 (95% CI: 0.52–0.70, *p* = 0.016), and specificity varied from 0.65 (95% CI: 0.58–0.71, *p* < 0.001) to 0.72 (95% CI: 0.67–0.77, *p* < 0.001).

### 3.5. Diagnostic Accuracy of Stress Echocardiography for Detecting Obstructive CAD

**Figure 5:**
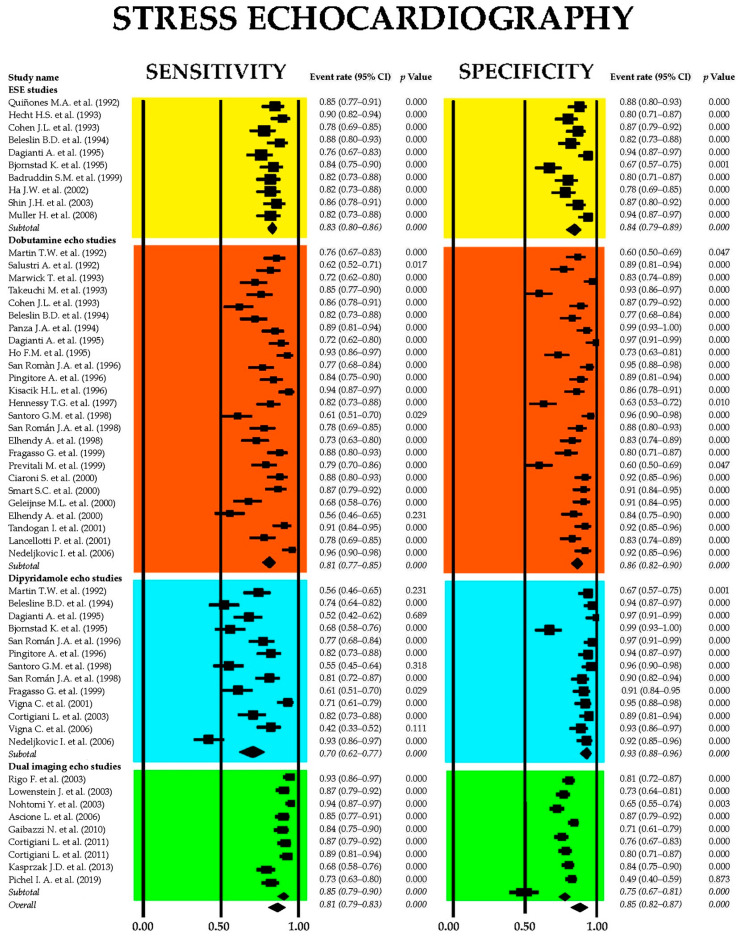
Forest plots illustrating the sensitivity and specificity of SE and its principal clinical applications, including ESE [51,52,53,54,55,56,57,58,59,60], DSE [26,28,29,30,33,36,37,44,53,54,55,61,62,63,64,65,66,67,68,69,70,71,72,73,74], dipSE [29,36,37,41,44,54,55,56,61,65,67,75,76], and dual-imaging SE [77,78,79,80,81,82,83,84]. DipSE, dipyridamole stress echocardiography; DSE, dobutamine stress echocardiography; ESE, exercise stress echocardiography; and SE, stress echocardiography.

Figure 5 displays the combined sensitivity and specificity estimates for SE and its primary clinical modalities, including ESE, dobutamine stress echocardiography (DSE), dipyridamole stress echocardiography (dipSE), and dual-imaging SE.

The aggregated sensitivity of SE for identifying obstructive CAD was 0.81 (95% CI: 0.79–0.83, *p* < 0.001), with a pooled specificity of 0.85 (95% CI: 0.82–0.87, *p* < 0.001). Among the various SE modalities, dipSE demonstrated the lowest sensitivity at 0.70 (95% CI: 0.62–0.77, *p* < 0.001), but the highest specificity at 0.93 (95% CI: 0.88–0.96, *p* < 0.001) for detecting CAD. The I^2^ statistic indicated significant heterogeneity, with values of 86.3% for sensitivity and 86.6% for specificity (both *p* < 0.001).

**Figure 6:**
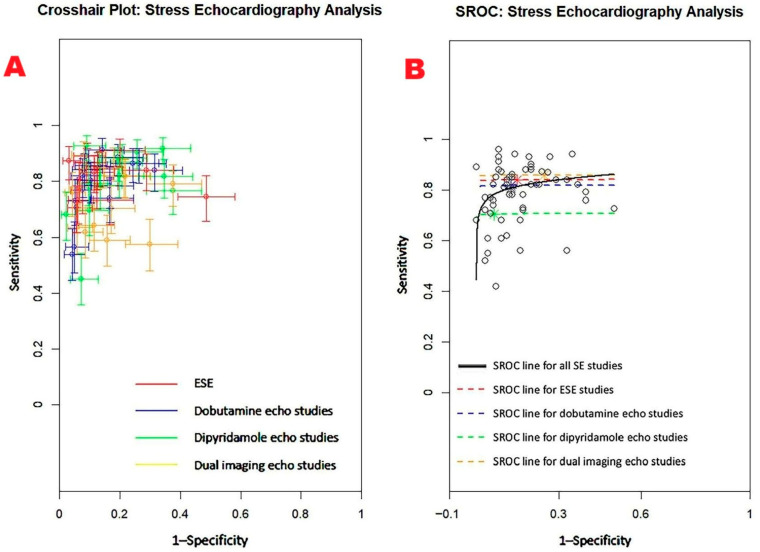
(**A**) Crosshair plot displaying the sensitivity and specificity results from studies evaluating various forms of stress echocardiography, including ESE, DSE, dipSE, and dual-imaging SE. (**B**) SROC curve illustrating the overall diagnostic accuracy of SE (AUC = 0.85; 95% CI: 0.80–0.91) and the individual performance of each technique: ESE (AUC = 0.84; 95% CI: 0.69–0.91), DSE (AUC = 0.82; 95% CI: 0.66–0.89), dipSE (AUC = 0.71; 95% CI: 0.46–0.86), and dual-imaging SE (AUC = 0.86; 95% CI: 0.75–0.91). DipSE, dipyridamole stress echocardiography; DSE, dobutamine stress echocardiography; ESE, exercise stress echocardiography; SE, stress echocardiography; and SROC, summary receiver operating characteristic.

Figure 6 presents (A) the crosshair plot illustrating sensitivity and specificity estimates for each SE modality—ESE, DSE, dipSE, and dual-imaging SE—and (B) the SROC curve, which integrates the diagnostic performance of overall SE and each individual technique.

**Figure 7:**
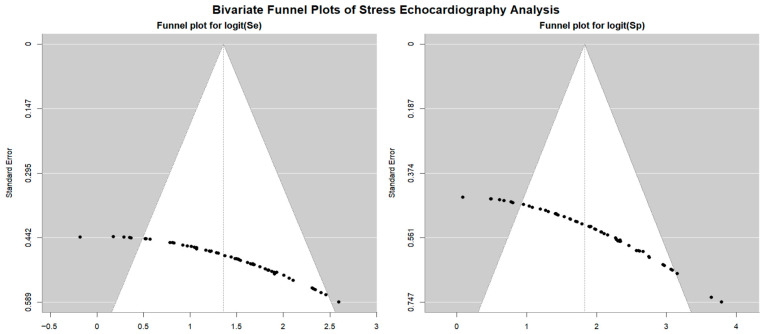
Begg’s funnel plot for the assessment of publication bias in studies evaluating sensitivity and specificity of SE for detecting obstructive CAD. CAD, coronary artery disease; SE, stress echocardiography.

Figure 7 displays the Begg’s funnel plot used to assess potential publication bias among the SE studies.

Egger’s generalized test revealed strong evidence of publication bias, with a *p* value of less than 0.001.

Sensitivity analyses reinforced the reliability of the overall findings. Stepwise exclusion of individual studies resulted in only minor fluctuations in sensitivity estimates across various stress echocardiography modalities. For ESE, sensitivity ranged from 0.83 (95% CI: 0.80–0.86, *p* < 0.001) to 0.87 (95% CI: 0.81–0.91, *p* < 0.001); in DSE studies, sensitivity varied from 0.69 (95% CI: 0.54–0.81, *p* = 0.01) to 0.82 (95% CI: 0.76–0.86, *p* < 0.001), while for dipSE studies, it ranged from 0.63 (95% CI: 0.52–0.72, *p* = 0.02) to 0.70 (95% CI: 0.63–0.76, *p* < 0.001). Finally, dual-imaging SE showed a sensitivity range between 0.87 (95% CI: 0.80–0.91, *p* < 0.001) and 0.91 (95% CI: 0.86–0.95, *p* < 0.001). Specificity estimates showed similar stability upon stepwise exclusion. For ESE, specificity ranged from 0.82 (95% CI: 0.76–0.87, *p* < 0.001) to 0.86 (95% CI: 0.81–0.90, *p* < 0.001); DSE studies reported specificity values from 0.88 (95% CI: 0.78–0.93, *p* < 0.001) down to 0.79 (95% CI: 0.58–0.91, *p* = 0.01), while for DipSE studies specificity ranged from 0.90 (95% CI: 0.61–0.98, *p* = 0.01) to 0.95 (95% CI: 0.85–0.98, *p* < 0.001). Finally, in dual-imaging SE studies specificity varied between 0.73 (95% CI: 0.63–0.81, *p* < 0.001) and 0.77 (95% CI: 0.66–0.85, *p* < 0.001).

### 3.6. Diagnostic Accuracy of Stress Myocardial SPECT for Predicting Obstructive CAD

**Figure 8:**
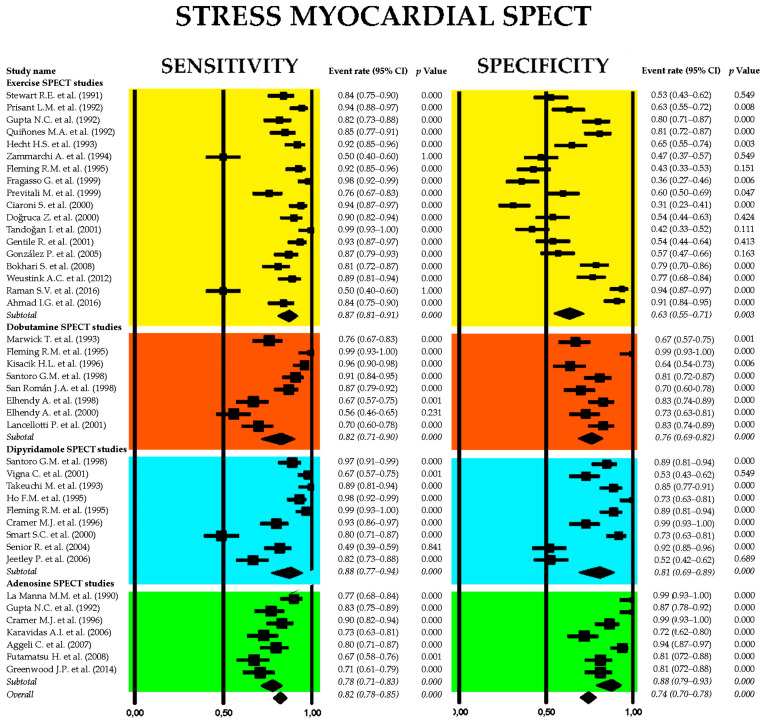
Forest plots showing the sensitivity and specificity of stress myocardial SPECT and principal stress myocardial scintigraphic techniques adopted in clinical practice: exercise [38,43,46,49,51,52,67,68,69,73,85,86,87,88,89,90,91,92], dobutamine [30,36,37,62,66,72,74,89], dipyridamole [36,63,64,70,76,89,94,95,96], and adenosine [87,94,97,98,99,100,101] myocardial SPECT. SPECT, single-photon emission computed tomography.

Figure 8 presents the combined sensitivity and specificity estimates for stress myocardial SPECT, including the main scintigraphic techniques commonly used in clinical settings—exercise, dobutamine, dipyridamole, and adenosine SPECT.

The pooled sensitivity of stress myocardial SPECT for detecting obstructive CAD was 0.82 (95% CI: 0.78–0.85, *p* < 0.001), with a pooled specificity of 0.74 (95% CI: 0.70–0.78, *p* < 0.001). Among the various stress scintigraphy methods, dipyridamole SPECT demonstrated the highest diagnostic performance, with a sensitivity of 0.88 (95% CI: 0.77–0.94, *p* < 0.001) and a specificity of 0.81 (95% CI: 0.69–0.89, *p* < 0.001). In contrast, exercise SPECT exhibited the lowest specificity at 0.63 (95% CI: 0.55–0.71, *p* = 0.003). Substantial heterogeneity was noted across the studies, with I^2^ values of 89.3% for sensitivity and 91.5% for specificity (both *p* < 0.001).

**Figure 9:**
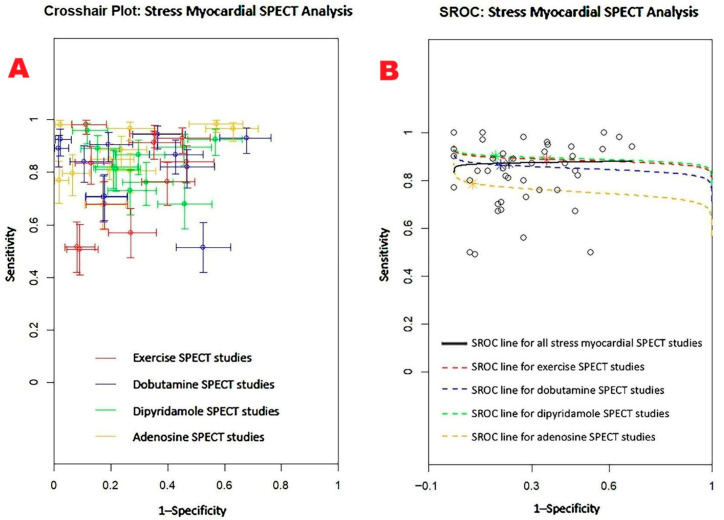
(**A**) Crosshair plot presenting the sensitivity and specificity results from studies evaluating stress myocardial SPECT using exercise, dobutamine, dipyridamole, and adenosine protocols. (**B**) SROC curve illustrating the overall diagnostic accuracy of stress myocardial SPECT (AUC = 0.88; 95% CI: 0.79–0.92), along with the performance of individual techniques: exercise SPECT (AUC = 0.88; 95% CI: 0.79–0.93), dobutamine SPECT (AUC = 0.85; 95% CI: 0.66–0.94), dipyridamole SPECT (AUC = 0.89; 95% CI: 0.73–0.95), and adenosine SPECT (AUC = 0.75; 95% CI: 0.47–0.91). SPECT, single-photon emission computed tomography; SROC, summary receiver operating characteristic.

Figure 9 displays the crosshair plot (A), highlighting the sensitivity and specificity results for individual SPECT modalities—including exercise, dobutamine, dipyridamole, and adenosine-based protocols. The SROC curve (B) provides an overview of the diagnostic performance of stress myocardial SPECT and its principal clinical techniques.

**Figure 10:**
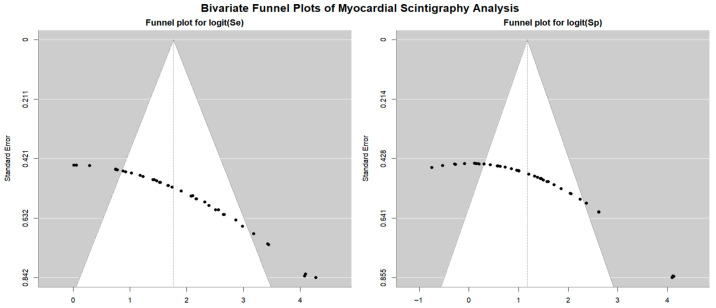
Begg’s funnel plot for the assessment of publication bias in studies evaluating sensitivity and specificity of stress myocardial SPECT for predicting obstructive CAD. CAD, coronary artery disease; SPECT, single-photon emission computed tomography.

Begg’s funnel plot for the detection of publication bias in stress myocardial SPECT studies is depicted in Figure 10.

Egger’s generalized test indicated significant publication bias, with a *p* value of less than 0.001. The sensitivity analyses substantiated the overall stability of the results. The sequential exclusion of individual studies produced only negligible fluctuations in sensitivity across the different myocardial SPECT modalities. For exercise SPECT, sensitivity ranged from 0.84 (95% CI: 0.69–0.92, *p* < 0.001) to 0.90 (95% CI: 0.74–0.96, *p* < 0.001); for dobutamine SPECT, it fluctuated between 0.85 (95% CI: 0.72–0.92, *p* < 0.001) and 0.94 (95% CI: 0.70–0.99, *p* = 0.005); dipyridamole SPECT sensitivity varied from 0.90 (95% CI: 0.78–0.96, *p* < 0.001) to 0.97 (95% CI: 0.84–0.99, *p* < 0.001); and adenosine SPECT showed a range from 0.79 (95% CI: 0.72–0.84, *p* < 0.001) to 0.84 (95% CI: 0.75–0.90, *p* < 0.001). Specificity values also showed minimal variation upon sequential study removal. For exercise SPECT, specificity ranged from 0.84 (95% CI: 0.69–0.92, *p* < 0.001) to 0.90 (95% CI: 0.74–0.96, *p* < 0.001); dobutamine SPECT ranged from 0.73 (95% CI: 0.62–0.82, *p* < 0.001) to 0.76 (95% CI: 0.61–0.86, *p* < 0.001); dipyridamole SPECT showed a range from 0.83 (95% CI: 0.72–0.91, *p* < 0.001) to 0.87 (95% CI: 0.77–0.93, *p* < 0.001); and adenosine SPECT specificity varied between 0.90 (95% CI: 0.80–0.95, *p* < 0.001) and 0.98 (95% CI: 0.76–0.99, *p* = 0.006).

### 3.7. Diagnostic Accuracy of Stress CMR for Detecting Obstructive CAD

**Figure 11:**
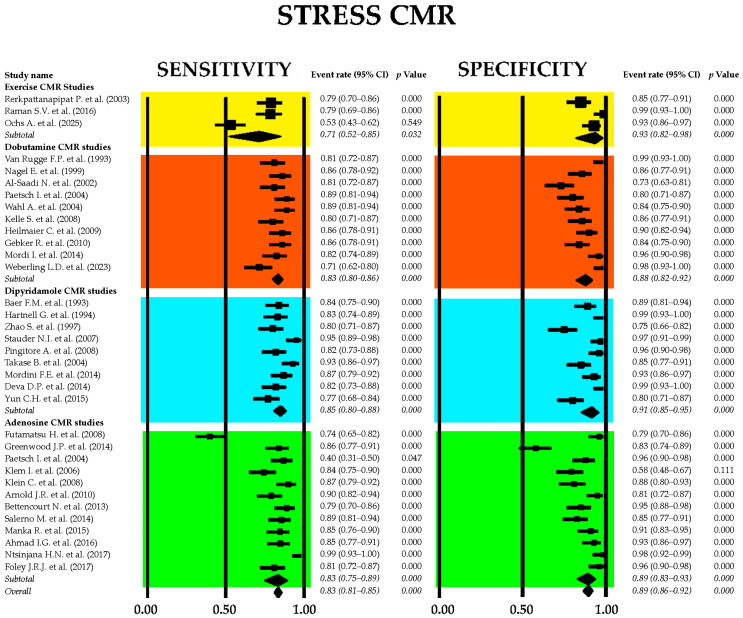
Forest plots showing the sensitivity and specificity of stress CMR and principal stress CMR methods used in clinical practice: exercise [91,102,103], dobutamine [27,104,105,106,107,108,109,110,111,112], dipyridamole [113,114,115,116,117,118,119,120,121], and adenosine [92,100,101,106,122,123,124,125,126,127,128,129] CMR. CMR, cardiac magnetic resonance.

Figure 11 displays the combined sensitivity and specificity values for stress CMR, including the primary techniques commonly employed in clinical settings—exercise, dobutamine, dipyridamole, and adenosine CMR.

The combined sensitivity of stress CMR imaging for detecting obstructive CAD was 0.83 (95% CI: 0.81–0.85, *p* < 0.001), while the overall specificity was 0.89 (95% CI: 0.86–0.92, *p* < 0.001). Among the different stress CMR modalities, dipyridamole-based CMR exhibited the highest sensitivity at 0.85 (95% CI: 0.80–0.88, *p* < 0.001), whereas exercise CMR demonstrated the greatest specificity, reaching 0.93 (95% CI: 0.82–0.98, *p* < 0.001). Significant heterogeneity was observed among the included studies, with I^2^ values of 83.0% for sensitivity and 82.5% for specificity (both *p* < 0.001).

**Figure 12:**
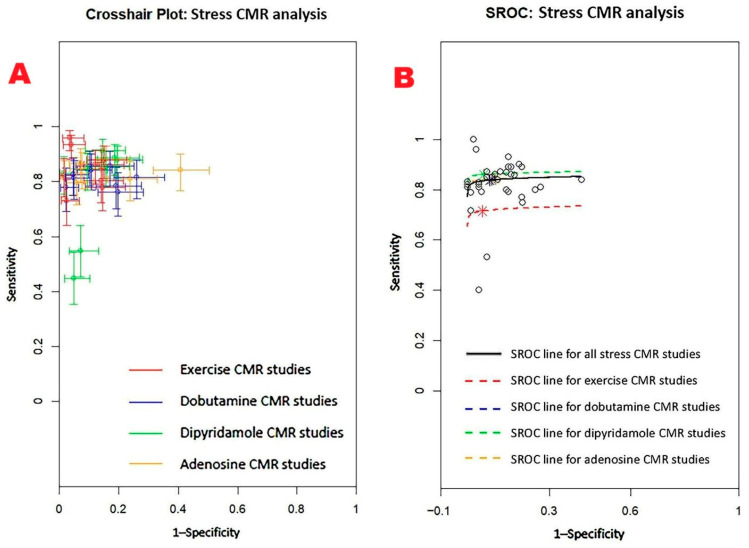
(**A**) Crosshair plot for stress CMR studies assessing sensitivity and specificity of exercise CMT, dobutamine CMR, dipyridamole CMR, and adenosine CMR. (**B**) SROC summarizing the overall performance of stress CMR (AUC = 0.85; 95%CI 0.60–0.94) and of each stress CMR technique: exercise (AUC = 0.74; 95%CI 0.30–0.95), dobutamine (AUC = 0.85; 95%CI 0.55–0.95), dipyridamole (AUC = 0.87; 95%CI 0.53–0.97), and adenosine (AUC = 0.85; 95%CI 0.57–0.95) CMR. CMR, cardiac magnetic resonance; SROC, summary receiver operating characteristic.

Figure 12 displays (A) the crosshair plot summarizing sensitivity and specificity data for each stress CMR method—exercise, dobutamine, dipyridamole, and adenosine—and (B) the SROC curve reflecting the overall diagnostic performance of stress CMR and its primary clinical applications.

**Figure 13:**
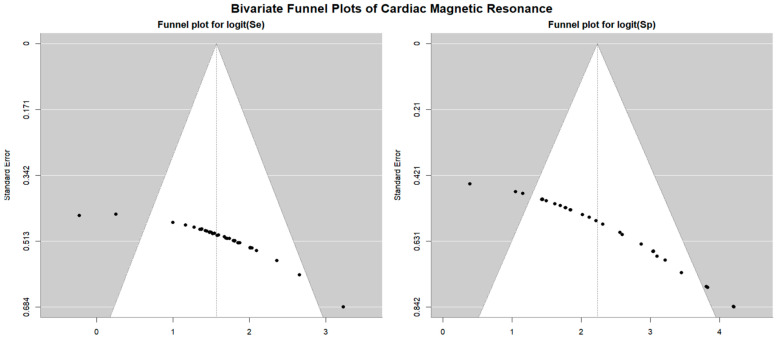
Begg’s funnel plot for the assessment of publication bias in studies evaluating sensitivity and specificity of stress CMR for identifying obstructive CAD. CAD, coronary artery disease; CMR, cardiac magnetic resonance.

The Begg’s funnel plot for the detection of publication bias in stress CMR studies is depicted in Figure 13.

Egger’s generalized test indicated significant publication bias with a *p* value less than 0.001. The sensitivity analysis affirmed the robustness of the results, with the sequential removal of individual studies yielding only minimal variations in sensitivity among the different CMR stress techniques. For dobutamine CMR, sensitivity ranged from 0.83 (95% CI: 0.78–0.86, *p* < 0.001) to 0.85 (95% CI: 0.81–0.88, *p* < 0.001); for dipyridamole CMR, from 0.82 (95% CI: 0.77–0.86, *p* < 0.001) to 0.86 (95% CI: 0.81–0.89, *p* < 0.001); and for adenosine CMR, from 0.78 (95% CI: 0.57–0.90, *p* = 0.01) to 0.83 (95% CI: 0.74–0.90, *p* < 0.001). Specificity estimates also varied slightly with study exclusion. For dobutamine CMR, specificity ranged from 0.83 (95% CI: 0.71–0.90, *p* < 0.001) to 0.86 (95% CI: 0.68–0.95, *p* = 0.001); for dipyridamole CMR, from 0.89 (95% CI: 0.70–0.97, *p* = 0.001) to 0.97 (95% CI: 0.58–1.00, *p* = 0.03); and for adenosine CMR, from 0.83 (95% CI: 0.69–0.91, *p* < 0.001) to 0.88 (95% CI: 0.81–0.93, *p* < 0.001).

### 3.8. Posterior Analysis of Sensitivity and Specificity of Methods

**Figure 14:**
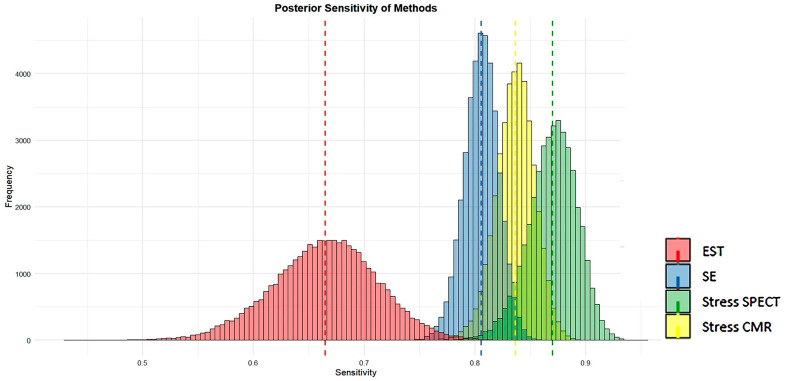
Posterior estimates of sensitivity of the four methods analyzed (EST, SE, stress SPECT, and stress CMR) for detecting obstructive CAD. CAD, coronary artery disease; CMR, cardiac magnetic resonance; EST, exercise stress testing; SE, stress echocardiography; and SPECT, single-photon emission computed tomography.

**Figure 15:**
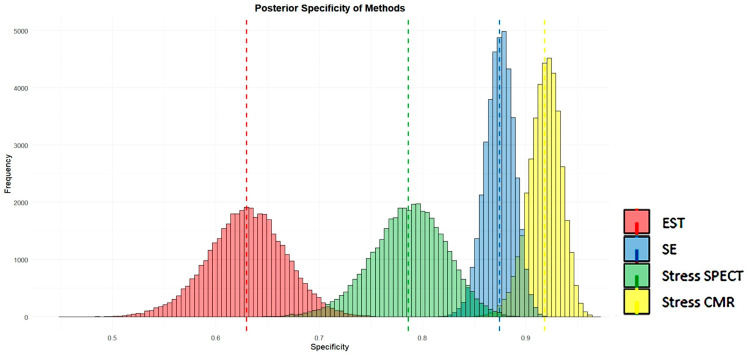
Posterior estimates of specificity of the four methods analyzed (EST, SE, stress SPECT, and stress CMR) for detecting obstructive CAD. CAD, coronary artery disease; CMR, cardiac magnetic resonance; EST, exercise stress testing; SE, stress echocardiography; and SPECT, single-photon emission computed tomography.

Figure 14 and Figure 15 visually present the posterior sensitivity and specificity estimates for the four evaluated techniques—EST, SE, stress SPECT, and stress CMR—in the diagnosis of obstructive CAD.

Figure 14 and Figure 15 show that SE and stress CMR exhibit high sensitivity and specificity in identifying CAD, with minimal variability as indicated by their narrow histogram distributions. In contrast, EST showed the lowest sensitivity and specificity accompanied by greater data dispersion, as reflected in its broader histograms. Unlike the other methods, stress myocardial SPECT displayed high sensitivity but comparatively lower specificity for detecting CAD. Overall, both stress CMR and SE outperformed SPECT and EST in terms of diagnostic accuracy.

### 3.9. Subgroup Analyses by Region, Study Period, and Study Design (By Modality)

The pooled, sample-size-weighted sensitivity and specificity for detecting obstructive CAD were also estimated by geographic region, by study period (1990–2004 vs. 2005–2025), and by study design (prospective vs. retrospective). These results are presented in Supplementary Tables S2–S4. While absolute values varied somewhat across subgroups, the relative ranking of modalities (CMR highest, SE intermediate, SPECT intermediate, and EST lowest) was consistent, supporting the robustness of the overall findings.

## 4. Discussion

### 4.1. Principal Findings of These Systematic Reviews and Meta-Analyses

These meta-analyses compared the diagnostic performance of four non-invasive stress testing modalities—EST, SE, stress myocardial SPECT, and stress CMR—for detecting obstructive CAD. EST demonstrated the lowest diagnostic accuracy, with a pooled sensitivity and specificity of 0.66 and 0.61, respectively; subgroup analysis revealed particularly low and statistically non-significant specificity in female-only studies. In contrast, SE achieved higher diagnostic values, showing a pooled sensitivity of 0.81 and specificity of 0.85. Among SE subtypes, dipSE had the lowest sensitivity (0.70) but the highest specificity (0.93), while dual-imaging SE demonstrated both high sensitivity and specificity. Stress myocardial SPECT exhibited a pooled sensitivity of 0.82 and specificity of 0.74; dipyridamole-based SPECT achieved the best performance among scintigraphic techniques, with a sensitivity and specificity of 0.88 and 0.81, respectively, while exercise SPECT had the lowest specificity (0.63). Stress CMR yielded the highest overall diagnostic accuracy, with a pooled sensitivity of 0.83 and specificity of 0.89; dipyridamole CMR provided the highest sensitivity (0.85), and exercise CMR achieved the best specificity (0.93).

Visual assessments supported these numerical findings. SE and CMR demonstrated strong sensitivity and specificity in detecting CAD, with limited variability, as evidenced by the narrow spread of its histogram distributions. Conversely, EST yielded the lowest sensitivity and specificity, along with increased data variability, as indicated by its wider histograms. Myocardial SPECT showed high sensitivity but comparatively lower specificity. Overall, stress SE and CMR were the most accurate techniques for detecting obstructive CAD, clearly outperforming both EST and myocardial SPECT in terms of diagnostic efficacy.

High heterogeneity was observed across the included studies, with I^2^ values ranging from 82.5% to 92.5%. This substantial between-study variability is likely attributable to differences in study region, time period, and design. Despite this heterogeneity, our additional subgroup analyses confirmed the relative ranking of diagnostic modalities: CMR demonstrated the highest accuracy, SE and SPECT showed intermediate performance, and EST consistently ranked lowest.

Egger’s generalized test indicated a statistically significant presence of publication bias (*p* < 0.05 across all methods), suggesting that smaller studies tended to report more favorable results, potentially inflating pooled accuracy estimates. Nevertheless, the relative ranking of modalities remained stable, reinforcing the robustness of the comparative findings.

Funnel plot analysis further revealed asymmetry in both sensitivity and specificity across all methods, suggesting that studies with lower accuracy were underrepresented. This underrepresentation may have contributed to overly optimistic pooled estimates, a phenomenon frequently observed in diagnostic accuracy meta-analyses. Despite these concerns, sensitivity analyses confirmed the overall robustness and reliability of the pooled results.

### 4.2. Factors Influencing the Diagnostic Accuracy of EST for Identifying Obstructive CAD

Evidence from published studies suggests that multiple variables can influence both the sensitivity and specificity of EST in diagnosing obstructive CAD. Sensitivity tends to decrease when myocardial stress is inadequate—often due to insufficient exercise duration or suboptimal exertion levels. This is commonly observed in women, individuals with reduced exercise capacity, patients on medications such as β-blockers, nitrates, or calcium channel blockers that blunt heart rate response and mask ischemic changes, or when ECG monitoring is limited [130,131,132,133]. It is also worth noting that ST-segment depression in inferior limb leads is less predictive of CAD than in lateral precordial leads (V4–V6) [46]. Additionally, caffeine intake before testing may lead to false negatives, as it interferes with adenosine-mediated vasodilation, thus impairing hyperemic response [134]. Left ventricular dysfunction may prevent an observable ischemic response during exercise, also yielding a false-negative result [135]. Other causes of a false-negative EST include male sex, single-vessel disease, involvement of the right coronary or circumflex artery, the presence of serial stenoses, or well-developed collateral circulation, all of which may prevent typical ECG changes despite underlying disease [49,136]. Posterior wall ischemia may be missed if the involved vessel is not adequately represented on standard ECG leads [137]. Similarly, in cases of multivessel disease, widespread but balanced underperfusion may not generate detectable ECG abnormalities [138].

On the other hand, several factors can compromise the specificity of EST. A hypertensive response during exercise is a commonly cited cause of false-positive results [58,139,140,141]. This response may indicate underlying left ventricular hypertrophy (LVH), which is associated with reduced coronary vasodilator reserve and subendocardial ischemia, even in the absence of atherosclerosis [140,141]. Some studies propose that a pronounced increase in systolic blood pressure during exercise may affect atrial repolarization [140,142]. Repeating the test after optimizing blood pressure control has been suggested as a useful strategy in such cases [58]. Female sex is another well-established predictor of false-positive EST results, with women demonstrating higher false-positive rates than men [49,131,132,133,143,144]. Explanations include lower pre-test probability, more significant heart rate increase, and a stronger hypertensive response to exercise [49,131,132,133,144]. Estrogen therapy has also been linked to increased false-positive rates, potentially due to estrogen’s structural similarity to digitalis [145] and its vasoconstrictive effects on coronary arterioles [32,39,133,146]. Other clinical conditions—such as severe valvular disease, mitral valve prolapse, cardiomyopathies, anemia, hypokalemia, arrhythmias, conduction disturbances (e.g., left bundle branch block, pacemaker rhythm, or Wolff–Parkinson–White syndrome), LVH, and digitalis therapy—can cause baseline ECG abnormalities that may be misinterpreted as ischemia during EST [35,47,49,133]. Furthermore, heightened myocardial sensitivity to catecholamines may result in heart rate elevations that mimic ischemic patterns despite normal coronary arteries [147]. A quick return of the ST segment to baseline (within one minute of recovery) often indicates a false-positive result, whereas persistent ST changes are more suggestive of true ischemia [139,148]. Finally, recent studies from our group have shown a link between chest wall deformities—such as a concave chest shape—and false-positive EST findings in symptomatic patients evaluated for suspected CAD [149,150]. This may be due to mechanical effects from the abnormal chest anatomy, potentially causing exercise-induced ECG alterations through cardiac compression, displacement, or electrical dyssynchrony.

Table 2 provides a summary of the various factors that may influence the sensitivity and specificity of EST in the detection of obstructive CAD.

### 4.3. Diagnostic Accuracy of Stress Echocardiography Modalities in Detecting Obstructive CAD

Stress echocardiography has evolved into a cornerstone of non-invasive diagnostic testing for CAD, allowing clinicians to assess inducible ischemia by observing changes in myocardial wall motion under stress. The four principal modalities—ESE, DSE, DipSE, and dual-imaging SE—each have distinct mechanisms, advantages, limitations, and diagnostic profiles.

ESE offers a physiologic and non-invasive method for diagnosing CAD by provoking ischemia through increased myocardial oxygen demand during physical exertion. One of its major advantages lies in its ability to simulate real-life cardiac stress, particularly useful in patients with normal exercise tolerance and interpretable electrocardiograms. ESE has shown a high specificity and a moderate-to-high sensitivity, especially in multivessel and left anterior descending (LAD) territory disease. In comparative studies, ESE and SPECT were concordant in approximately 88% of cases, and when used in combination, sensitivity improved, although at the cost of reduced specificity [51]. While SPECT evaluates perfusion and echocardiography evaluates wall motion, both modalities detect ischemia in overlapping myocardial territories, each capturing abnormalities missed by the other. Notably, ESE demonstrated superior sensitivity for detecting LAD artery lesions and in patients with triple-vessel disease, especially when images were acquired at peak exercise rather than post-exercise [52]. Supine bicycle echocardiography, a variation of ESE, provides increased venous return and oxygen demand, enhancing ischemic burden and improving the visualization of wall motion abnormalities [57]. Despite these strengths, ESE faces several limitations. The accuracy of the test is highly operator-dependent, relying on both the technical skill of the sonographer and the interpretative expertise of the physician. Imaging during exercise can be technically difficult due to hyperventilation and excessive chest wall movement, often necessitating apical-only views and potentially leading to false interpretations [151]. Furthermore, submaximal stress from inadequate exercise capacity, particularly in elderly, hypertensive, or deconditioned patients, may yield non-diagnostic or false-negative results [60]. False-positive results are more frequent in patients with a hypertensive response to exercise, where increased afterload and wall stress may mimic ischemia without significant epicardial stenosis [58]. Women are also more prone to false positives due to smaller heart size, breast attenuation, and apical thinning, although test specificity becomes similar to that of men when verification bias is adjusted [152]. Overall, while ESE is physiologic, widely available, and cost-effective, its limitations in patient subgroups and imaging artifacts must be acknowledged.

DSE serves as a valuable alternative in patients unable to exercise. It increases heart rate, blood pressure, and myocardial contractility, thereby provoking ischemia through elevated oxygen demand [153]. Compared to ESE, DSE has slightly higher sensitivity for detecting CAD, particularly in one-vessel disease and when imaging is technically limited during exercise [53]. It is especially effective in detecting severe and multivessel CAD, with the highest sensitivity reported in patients with stenosis exceeding 70% [154]. Additionally, DSE has the benefit of fewer motion-related artifacts, and ischemia tends to occur at a lower heart rate, enhancing early detection. The diagnostic accuracy of DSE can be further improved by co-administering atropine, particularly in patients on beta-blockers or those with an inadequate chronotropic response [155,156]. Transesophageal DSE can also be used in patients with poor transthoracic acoustic windows, such as the obese, elderly, or post-surgical patients, offering excellent imaging quality and accuracy for LAD and right coronary artery (RCA) territory disease [28]. Nevertheless, DSE has its own limitations. It is less specific than dipyridamole echocardiography and may provoke arrhythmias, including atrial fibrillation and supraventricular tachycardia [65,157]. False positives may occur in non-ischemic cardiomyopathies and patients with resting wall motion abnormalities, particularly in the basal segments [33]. DSE may also underestimate CAD in cases of mild or single-vessel disease and in submaximal tests where side effects limit the full dose [63]. Additionally, DSE has a lower sensitivity in detecting lesions in RCA or left circumflex (LCx) compared to LAD due to the smaller myocardial mass they perfuse [72]. Despite this, DSE remains one of the most widely validated pharmacological stress tests, with a diagnostic accuracy comparable to perfusion scintigraphy, and superior to EST [30].

DipSE, a vasodilator-based modality, is another option for patients unable to exercise. It induces ischemia by increasing coronary flow and creating a subendocardial steal in territories supplied by stenotic arteries. DipSE’s chief advantage is its very high specificity, particularly for multivessel disease, and its safety profile is favorable, with fewer arrhythmic complications than DSE [158]. In hypertensive patients and those with left bundle branch block (LBBB) or pacemakers, DipSE maintains high specificity, outperforming perfusion scintigraphy in these subgroups [69,73]. The test is further enhanced by adding atropine to increase heart rate and sensitivity, especially in patients under beta-blocker therapy [156,159]. Imaging quality is usually superior due to stable hemodynamics and minimal patient motion. However, DipSE is limited by its lower sensitivity, particularly in detecting single-vessel or mild CAD [160,161]. Its effectiveness may also be blunted by concurrent antianginal therapy, which prevents sufficient coronary steal to induce ischemia [54]. Additionally, DipSE primarily affects coronary supply and may not produce the myocardial oxygen mismatch seen with exercise or dobutamine, making wall motion abnormalities less frequent. False negatives are thus more likely when ischemic burden is mild or confined to small myocardial regions [29]. While technically simpler and better tolerated, its diagnostic yield is best in more advanced disease or when combined with other diagnostic approaches.

Dual-imaging SE, typically combining wall motion assessment with coronary flow reserve (CFR) measured via Doppler, represents a novel approach to improving diagnostic sensitivity. The integration of CFR, particularly in the LAD territory, adds significant value in cases where wall motion appears normal but microvascular or early epicardial disease is suspected [162]. The method enhances the detection of mild or single-vessel CAD and improves risk stratification, especially in hypertensive patients with subclinical microvascular dysfunction [82,163]. A normal CFR has high negative predictive value, while an abnormal CFR often precedes detectable wall motion abnormalities, thereby increasing sensitivity [83,164]. The combined analysis of wall motion and CFR outperforms either method alone, and may approach the diagnostic yield of myocardial perfusion imaging, without radiation exposure [81]. Despite its promise, dual-imaging has technical challenges. The acquisition of CFR via transthoracic Doppler requires significant expertise, is time-consuming, and is often limited to the LAD due to anatomical constraints [79]. Furthermore, CFR cannot distinguish between microvascular and macrovascular disease, and its specificity may be reduced in patients with diabetes, hypertension, or hypercholesterolemia, who may have microvascular dysfunction in the absence of epicardial stenosis [165,166,167]. Nevertheless, in selected patients, particularly those with an intermediate likelihood of disease or suboptimal wall motion analysis, the dual-imaging strategy offers a powerful diagnostic and prognostic tool.

Table 3 summarizes the main advantages and technical limitations of ESE, DSE, DipSE, and dual-imaging stress echocardiography for CAD detection in clinical practice.

### 4.4. Diagnostic Accuracy of Stress Myocardial SPECT for CAD Detection

Myocardial perfusion imaging with SPECT remains a cornerstone in the non-invasive diagnosis of CAD. The stress modality used—whether physiological (exercise) or pharmacologic (dobutamine, dipyridamole, or adenosine)—significantly influences diagnostic accuracy, tolerance, and image quality. Each modality presents unique advantages and limitations.

Exercise stress SPECT is a physiological method that provides valuable functional information in addition to perfusion assessment. It has demonstrated superior diagnostic performance compared to EST in detecting CAD in both single- and multivessel disease [46]. Its ability to assess physical capacity also adds prognostic value. Moreover, perfusion abnormalities often occur earlier than ischemia detectable by ECG, which enhances sensitivity [168]. However, about 25–30% of patients may not achieve adequate exercise levels due to comorbidities [169]. Attenuation artifacts—particularly diaphragmatic attenuation in men and breast attenuation in women—can result in high false-positive rates, notably affecting the inferior and anterior walls [88,170]. LBBB is another major limitation, as it leads to septal perfusion defects in the absence of CAD [73].

Dobutamine stress SPECT is indicated when exercise is not feasible. It increases myocardial oxygen demand by enhancing heart rate and contractility, mimicking exercise. It has shown very high sensitivity and specificity in limited cohorts [89], particularly for detecting single-vessel CAD and LAD lesions. It is preferred in patients with LBBB or LVH, where perfusion SPECT may yield false positives [62]. Nonetheless, dobutamine may cause pro-arrhythmia, and its uptake may interfere with MIBI imaging in normally perfused areas, potentially leading to an underestimation of flow heterogeneity [171]. The diagnostic performance of dobutamine SPECT is comparable to stress echocardiography, although the latter may have higher specificity in certain subgroups.

Dipyridamole stress SPECT offers a non-exertional alternative by inducing coronary vasodilation. It has shown high sensitivity in CAD detection, particularly in multivessel disease [36]. Its diagnostic efficacy is comparable to that of adenosine, with good agreement in terms of regional tracer uptake and hemodynamic effects [94]. Dipyridamole is especially useful in detecting perfusion abnormalities in territories with mild-to-moderate stenosis, which may not produce echocardiographic changes [172]. However, its specificity is modest due to attenuation artifacts and motion-related false positives [173]. Though side effects like flushing or chest discomfort are common, they are generally reversible with aminophylline.

Adenosine stress SPECT is widely used due to its short half-life, rapid onset of action, and safety profile. Like dipyridamole, it produces vasodilation but with a shorter infusion time and reduced potential for bronchospasm. The diagnostic accuracy of adenosine SPECT is similar to that of exercise SPECT in detecting both fixed and reversible perfusion defects [97]. It shows particular superiority in LBBB patients by minimizing false-positive septal defects [73,174]. Additionally, it has shown higher sensitivity than dobutamine for perfusion imaging, though specificity may be lower in patients with a smaller heart size or single-vessel disease [101]. Continuous ECG and blood pressure monitoring are recommended due to transient side effects such as chest pain, atrioventricular block, or bradycardia [87].

The choice of stress modality in myocardial SPECT should be individualized based on patient characteristics, ability to exercise, comorbidities (e.g., LBBB and LVH), and institutional expertise. Exercise remains the preferred first-line option when feasible, while pharmacologic stressors offer robust alternatives with comparable diagnostic power. Nonetheless, each carries unique artifacts and side effect profiles, influencing diagnostic interpretation and clinical decision-making.

Table 4 summarizes the main advantages and limitations of exercise stress SPECT, dobutamine stress SPECT, dipyridamole stress SPECT, and adenosine stress SPECT for CAD detection.

### 4.5. Diagnostic Accuracy of Stress CMR for Detecting Obstructive CAD

CMR stress testing has evolved into a powerful non-invasive tool for detecting CAD, utilizing various stressors including exercise, dobutamine, dipyridamole, and adenosine. Exercise stress CMR, though technically demanding, offers physiological relevance by simulating natural exertion. Early studies using treadmill-based protocols demonstrated promising diagnostic accuracy, with sensitivity and specificity reported at 79% and 85%, respectively, for detecting significant coronary stenoses [102]. A larger multicenter study confirmed excellent correlation with angiographic findings and outperformed SPECT in diagnostic performance, with a sensitivity and specificity of 79% and 99%, respectively [91]. Exercise CMR can also identify microvascular dysfunction [175]. However, its clinical adoption has been limited due to logistical hurdles such as delayed imaging after exercise, which may diminish the detection of transient ischemic changes. Supine bicycle ergometry is feasible but often hampered by muscle fatigue in untrained patients. Recently, dynamic handgrip exercise combined with fast strain–encoded imaging (DHE–fSENC) emerged as a viable, shorter, and more accessible stress protocol, showing a sensitivity and specificity of 82% and 89% for obstructive CAD, even in elderly populations [103].

Dobutamine stress cardiac magnetic resonance (DSCMR), a pharmacologic alternative, mimics exercise by increasing heart rate and myocardial oxygen demand. It is well established as a feasible, safe, and highly accurate method for CAD detection, with reported sensitivity and specificity ranging from 81 to 89% and 85 to 100%, respectively [27,109]. Compared to DSE, DSCMR has shown superior diagnostic accuracy due to better spatial resolution, clearer endocardial border delineation, and reduced operator dependency [104]. Technical advances such as steady-state free precession sequences and parallel imaging have further enhanced image quality [176]. DSCMR has proven effective across various subgroups, including patients with multivessel disease, prior stenting, and chronic kidney disease [177]. Its negative predictive value remains high (95%), supporting its utility in ruling out significant CAD [112]. However, limitations include reduced accessibility and limited applicability in patients with claustrophobia or non-MR-compatible devices. Additional concerns involve suboptimal temporal resolution—particularly at high heart rates—and the inability to interpret diagnostic ECGs due to magnetic interference.

Dipyridamole stress CMR primarily assesses perfusion and wall motion abnormalities. Studies have demonstrated comparable diagnostic accuracy to nuclear imaging, with sensitivity and specificity frequently exceeding 85% [113,116]. It is especially sensitive in detecting moderate stenoses via perfusion deficits and more specific for severe disease using wall motion criteria [118]. Dipyridamole-induced perfusion defects correlate strongly with significant CAD and prior myocardial infarction. A fully quantitative perfusion method outperformed both semi-quantitative and qualitative analyses, achieving an AUC of 92%, sensitivity of 87%, and specificity of 93% [119]. This modality is safe and effective even in complex scenarios such as congenital heart disease [120] or post-bypass graft evaluation [117], and offers utility in comprehensive assessments combining cine, perfusion, and LGE imaging.

Adenosine stress CMR is now widely used and regarded for its rapid infusion protocol, high safety profile, and excellent spatial resolution. It offers good sensitivity (84–89%) and specificity (87–98%) for detecting functionally significant CAD, especially when combined with late gadolinium enhancement (LGE) [122]. The multiparametric approach of adenosine perfusion-CMR, incorporating rest perfusion, cine imaging, and LGE, improves specificity and helps differentiate artifacts from true ischemia. While artifacts like dark-rim effects may reduce specificity, innovations such as spiral pulse sequences have minimized their impact [126]. Adenosine stress CMR performs robustly at 3.0T, showing excellent accuracy in both pediatric and adult populations [127,128], and is superior to SPECT, particularly in women and overweight patients, due to higher spatial resolution and reduced attenuation artifacts [101].

Table 5 lists the relevant advantages and limitations of exercise stress CMR, dobutamine stress CMR, dipyridamole, stress CMR, and adenosine stress CMR for predicting obstructive CAD.

### 4.6. Clinical Applicability of EST, SE, SPECT, and CMR

In clinical practice, the choice among EST, SE, SPECT, and CMR must balance diagnostic accuracy with availability, patient safety, and resource considerations. EST remains the most widely available and least expensive technique, requiring minimal infrastructure; however, its diagnostic yield is limited, particularly in women, elderly patients, and those unable to achieve adequate exercise capacity. For this reason, its role has shifted toward functional capacity assessment and symptom reproduction rather than the primary diagnosis of obstructive CAD. SE offers an attractive compromise: it is broadly available, relatively inexpensive compared with advanced imaging, and does not involve radiation exposure. Operator expertise is essential, but when performed well, SE achieves robust accuracy and can be integrated into routine workflows. SPECT is still the most common imaging test worldwide, with extensive evidence and standardized protocols. It provides reproducible results and broad accessibility, yet its limitations include radiation exposure, attenuation artifacts, and declining specificity in certain populations. CMR has emerged as the most accurate modality, offering high spatial resolution, no ionizing radiation, and a comprehensive assessment of structure, function, and perfusion. Nonetheless, its uptake is constrained by limited availability, longer examination times, contraindications in patients with devices or claustrophobia, and higher costs. Taken together, SE provides the most pragmatic balance of accuracy, safety, and feasibility, SPECT offers a familiar and standardized tool, CMR represents the accuracy benchmark where available, and EST, although inexpensive, has a marginal role in contemporary diagnostic pathways.

### 4.7. 2024 ESC Guidelines Recommendations for the Diagnostic Management of Patients with Suspected CAD

The 2024 European guidelines underscore the evolving role of diagnostic testing in patients with suspected chronic coronary syndrome (CCS), emphasizing the stratification of patients based on pre-test probability (PTP) and the appropriate use of non-invasive imaging modalities to diagnose obstructive CAD and estimate the risk of major adverse cardiovascular events (MACE).

EST retains value, particularly for patients with a low clinical likelihood of CAD (>5–15%), where a negative result can reclassify individuals into a very low risk group (≤5%) with a favorable prognosis [178]. It remains clinically relevant for reproducing anginal symptoms, which are themselves prognostic [179,180]. Traditionally, the EST involves graded physical activity until the appearance of limiting symptoms or abnormal ECG findings. While widely used, its diagnostic accuracy is inferior to modern functional imaging techniques and coronary computed tomography angiography (CCTA), with sensitivity and specificity reported at only 58% and 62%, respectively [181]. Data from the SCOT–HEART trial showed that abnormal EST results predict future revascularization and myocardial infarction, yet a large subset of patients with normal or inconclusive tests still had underlying CAD that was better detected with CCTA [182]. Consequently, CCTA is now preferred as a first-line diagnostic strategy, especially in individuals with low or moderate PTP. Nevertheless, EST may remain relevant in regions with limited access to imaging or as a pragmatic option for symptom evaluation and risk stratification. However, the test is not appropriate in patients with baseline ECG abnormalities, such as LBBB, paced rhythms, or digitalis therapy, which interfere with interpretation.

In individuals with moderate or high PTP of obstructive CAD (>15–85%), the guidelines recommend SE, SPECT, or positron emission tomography (PET) myocardial perfusion imaging, or stress CMR, as first-line tests. SE assesses myocardial ischemia by detecting regional wall-thickening abnormalities (RWTA), induced by increased oxygen demand during stress. The technique can utilize exercise, dobutamine, or vasodilators such as adenosine or dipyridamole. Though highly accessible, low-cost, and radiation-free [183,184], its effectiveness depends on operator skill and image quality, which may be compromised in patients with obesity or lung disease. RWTA-based diagnosis may underestimate ischemia in patients with microvascular dysfunction [185], making it less suitable for conditions like angina with non-obstructive coronary arteries (ANOCA) or ischemia with non-obstructive coronary arteries (INOCA). Additional stress echocardiography techniques, such as the measurement of CFR in the LAD artery, offer incremental value in risk stratification [186]. The use of ultrasound contrast agents (microbubbles) is also recommended when two or more myocardial segments are poorly visualized. SE is especially recommended in patients with moderate-to-high PTP to diagnose myocardial ischemia and estimate MACE risk [6].

Myocardial perfusion SPECT imaging evaluates regional myocardial blood flow using radiotracers, with technetium–99m preferred over thallium due to lower radiation exposure. Although traditionally limited by attenuation artifacts and reduced accuracy in multivessel CAD, newer cadmium–zinc telluride (CZT) detectors have improved its spatial resolution, reduced scan time, and enabled myocardial blood flow (MBF) quantification [187]. SPECT remains widely applicable, including for patients unable to exercise. In high-risk populations, it offers good diagnostic and prognostic utility [188,189,190,191]. When SPECT or PET is selected, coronary artery calcium scoring (CACS) from CT attenuation correction imaging is recommended for the enhanced detection of non-obstructive CAD.

Stress CMR, using gadolinium-enhanced perfusion imaging, offers high spatial resolution without radiation and provides a comprehensive evaluation including the assessment of LV function and myocardial scarring via LGE [181,192,193]. It is particularly suited to patients with suspected CAD who require an assessment of both ischemia and myocardial viability. Stress CMR has shown strong prognostic value [194,195,196] and enhances management decisions [191,197]. However, limitations include restricted availability, claustrophobia, longer acquisition times [198], and contraindications in patients with certain implants or advanced renal dysfunction.

Finally, CCTA is recommended as the first-line test in patients with low to moderate PTP (5–50%), based on its high negative predictive value and ability to detect non-obstructive CAD [199,200,201]. It is particularly advantageous in younger patients or those with atypical symptoms. In contrast, functional imaging is preferred when information on ischemia, viability, or microvascular disease is required, and when CCTA’s utility may be limited, such as in cases of severe coronary calcification, arrhythmias, or contrast allergies.

ESC 2024 recommendations regarding the use of EST, SE, stress myocardial SPECT, and stress CMR in clinical settings are outlined in Table 6.

### 4.8. Innovative Screening Methods for CAD Detection

In recent years, our research group has conducted several studies aimed at examining how chest wall conformation may impact the outcomes of EST and/or ESE. By incorporating a preliminary, noninvasive assessment of chest wall shape using the modified Haller index (MHI)—calculated as the ratio between the transverse external thoracic diameter and the anteroposterior internal thoracic diameter [202]—into PTP evaluation, we improved the identification of patients with a low or extremely low likelihood of CAD and/or an increased chance of a false positive EST or ESE result.

Specifically, a concave chest wall shape—often resulting from varying degrees of sternal depression or pectus excavatum and defined by an MHI greater than 2.5 [203]—was shown to independently predict false positive ESE outcomes [204] and was strongly linked to a favorable cardiovascular prognosis and a low risk of adverse events over mid-to-long-term follow-up [205,206]. Individuals with this chest conformation (MHI > 2.5) are typically women with a smaller body surface area and a lower incidence of traditional cardiovascular risk factors. These patients are frequently referred to echocardiographic labs to rule out CAD due to resting or stress ECG abnormalities.

Common findings in such patients include smaller cardiac chambers, preserved left ventricular geometry, normal left ventricular systolic and diastolic function, and a higher occurrence of mitral valve prolapse. Notably, mitral valve prolapse often coexists with anterior chest wall deformities and thoracic skeletal anomalies, including pectus excavatum, pectus carinatum, scoliosis, straight back syndrome, and Marfan syndrome [207]. Those with an MHI > 2.5 and/or mitral valve prolapse often report atypical chest pain and palpitations and tend to present with resting or exercise-induced premature ventricular contractions and pseudo-ischemic ECG patterns [208].

A reduced anteroposterior chest diameter (typically ≤13.5 cm) [204] may contribute to, or independently cause, dynamic LV dyssynchrony during physical exertion, driven by external compressive forces rather than intrinsic myocardial impairment or actual ischemia. Such dyssynchrony may be misinterpreted as regional wall motion abnormalities suggestive of obstructive CAD, even by experienced echocardiographers. Additionally, atrial and/or ventricular arrhythmias stemming from the sternal compression of cardiac structures are commonly observed in individuals with varying degrees of anterior chest wall deformity.

Clinically, these findings suggest that in symptomatic patients with a low PTP for CAD (<15%), an MHI > 2.5 or an anteroposterior chest diameter ≤ 13.5 cm, and mitral valve prolapse, a positive EST or ESE result is likely to be falsely positive. For such patients, it may be advisable to forego EST, ESE, or further imaging like CCTA or invasive coronary angiography. On the other hand, a circular or convex chest shape (MHI ≤ 2.5 or anteroposterior chest diameter > 13.5 cm) is often associated with enlarged cardiac chambers, atrial fibrillation, and CAD [209]. Therefore, symptomatic individuals with an intermediate-to-high PTP for CAD (15–85%) and MHI ≤ 2.5 warrant further diagnostic evaluation.

According to findings from this meta-analysis, SE (preferably ESE in patients capable of exercise) may serve as the preferred initial test for detecting CAD, given its high diagnostic performance, broad availability, and cost-effectiveness. A clearly negative SE can reliably exclude obstructive CAD. Conversely, a positive or inconclusive SE result may necessitate further imaging like CCTA. This diagnostic pathway may reduce unnecessary exposure to ionizing radiation from myocardial SPECT and may help avoid referrals to stress CMR, which is often less accessible due to its higher cost and time requirements.

**Figure 16:**
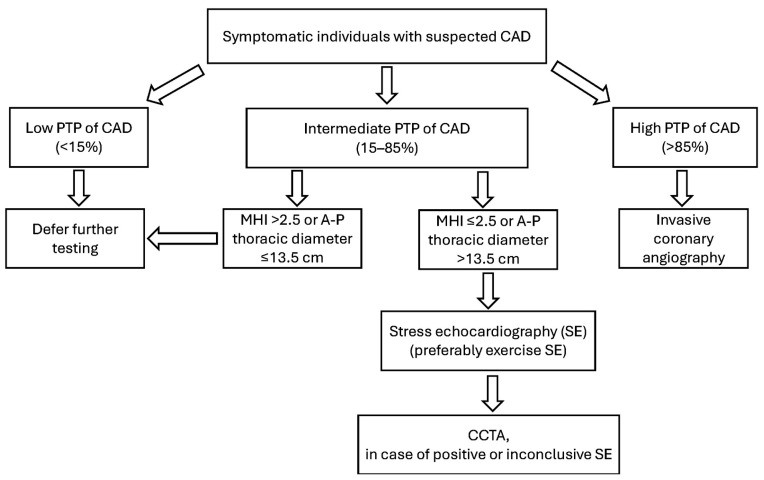
Innovative screening strategy for symptomatic individuals with suspected CAD. A–P, antero–posterior; CAD, coronary artery disease; CCTA, coronary computed tomography angiography; MHI, modified Haller index; PTP, pre-test probability; and SE, stress echocardiography.

A flowchart outlining this proposed screening strategy for CAD in symptomatic patients, stratified by PTP, is shown in Figure 16.

### 4.9. Limitations of the Studies Included in the Present Meta-Analysis

A key limitation of the studies included in this analysis was their generally small sample sizes and the fact that most were conducted at single centers. Additionally, 8.6% of the studies were retrospective in design. The I^2^ values for EST, SE, stress myocardial SPECT, and stress CMR studies ranged from 82.5% to 92.5%, reflecting substantial heterogeneity among the studies. This variability is partly attributable to differences in the populations studied—symptomatic patients from different countries, age groups, and sexes, with varying degrees of CAD severity. Approximately half of the studies enrolled patients with suspected CAD only, whereas the remainder included those with known CAD or mixed populations. Since diagnostic accuracy often appears higher in known CAD, this mixture may have biased the pooled estimates upward. In addition, the angiographic thresholds used to define obstructive CAD varied (≥50% vs. ≥70% stenosis), further limiting the direct comparability of results across studies. Furthermore, the studies used differing stress protocols, varying ischemic stressor doses, and distinct angiographic criteria for defining obstructive CAD. The presence of “small study effects”—where smaller trials (which made up a large proportion of this meta-analysis) tend to show more favorable or exaggerated outcomes compared to larger studies [210]—was evident; consequently, Egger’s generalized test indicated significant publication bias in the data sets for EST, SE, stress myocardial SPECT, and stress CMR. Nevertheless, sensitivity analyses affirmed the stability and reliability of the findings across all diagnostic meta-analyses. Finally, the present meta-analysis did not include studies assessing coronary microvascular dysfunction in patients with suspected CAD. In this regard, recent findings suggest that exercise-induced ischemic ECG alterations are highly specific for detecting coronary microvascular dysfunction in patients with ANOCA [211].

## 5. Conclusions

Among the diagnostic tools assessed, EST demonstrated the least effective diagnostic performance for identifying obstructive CAD, whereas stress CMR exhibited the highest overall accuracy. Although stress myocardial SPECT was characterized by strong sensitivity, its specificity was relatively limited. SE emerged as a favorable middle ground, offering a reliable balance of diagnostic accuracy and practical advantages, including broad accessibility, cost-efficiency, and the absence of radiation exposure.

## Figures and Tables

**Table 1:** Clinical characteristics, stress protocols, and main findings of the 159 total analyses assessing sensitivity, specificity, and accuracy of EST, SE, stress myocardial SPECT, and stress CMR for detecting obstructive CAD. Acc, accuracy; CAD, coronary artery disease; CFR, coronary flow reserve; CMR, cardiac magnetic resonance; ESE, exercise stress echocardiography; EST, exercise stress testing; IV, intravenous; Monoc., monocentric; NS, not specified; P., prospective; SE, stress echocardiography; Sen, sensitivity; Spe, specificity; SPECT, single-photon emission computed tomography; Tc, Technetium; and Tl, Thallium. For the population study of Cortigiani L. et al. [82], hypertensive * and normotensive ** patients were separately analyzed.

Author, Publication Year, Country	Study Design	Size (%fem)	Av. Age (y)	CAD History	Cut-Off for CAD	Stress Protocols (Ischemic Stressors Doses)	Sen (%)	Spe (%)	Acc (%)
**EST studies**									
Salustri A., 1992, The Netherlands [26]	P., monoc.	52 (26.9)	58	Suspected	≥50%	Upright bicycle exercise test (stepwise increments of 20 Watts every minute)	46	67	56.5
van Rugge F.P., 1993, The Netherlands [27]	P., monoc.	45 (20)	61	Suspected	≥50%	Standard Bruce protocol (stepwise increments of 25 Watts every 2 min)	70	63	69
Panza J.A., 1994, USA [28]	R, monoc.	76 (18.4)	60	Known or suspected	≥70%	Standard Bruce protocol (stepwise increments of 25 Watts every 2 min)	55	77	68
San Román J.A., 1996, Spain [29]	P., monoc.	102 (44.1)	62	Suspected	≥50%	Standard Bruce protocol (stepwise increments of 25 Watts every 2 min)	68	79	73.5
Kisacik H.L., 1996, Turkey [30]	P., monoc.	69 (15.9)	51	Known or suspected	≥50%	Standard Bruce protocol (stepwise increments of 25 Watts every 2 min)	60	64	61
Laurienzo J.M., 1997, USA [31]	P., monoc.	84 (100)	51	Known or suspected	≥70%	Standard Bruce protocol (stepwise increments of 25 Watts every 2 min)	76	68	70
Morise A.P., 1997, USA [32]	R, monoc.	781 (49)	54	Suspected	≥50%	NS	23	77	50
Hennessy T.G., 1997, Ireland [33]	P., monoc.	116 (29.3)	59	Suspected	≥50%	Standard Bruce protocol (stepwise increments of 25 Watts every 2 min)	40	79	59.5
Ho Y.L., 1998, China [34]	P., monoc.	51 (100)	62	Known or suspected	≥50%	Standard Bruce protocol (stepwise increments of 25 Watts every 2 min)	71	44	57
Kalaria V.G., 1998, USA [35]	P., multic.	882 (23)	57	Known	≥50%	Sheffield modification of Bruce protocol (3-minute “stage 1/2” before the Bruce stage I)	54	57	56
Santoro G.M., 1998, Italy [36]	P., monoc.	60 (NS)	NS	Suspected	≥70%	Upright bicycle exercise test (stepwise increments of 30 Watts every 3 min)	58	67	62
San Román J.A., 1998, Spain [37]	P., monoc.	102 (51)	64	Suspected	≥50%	Standard Bruce protocol (stepwise increments of 25 Watts every 2 min)	66	80	70
Gentile R., 2001, Italy [38]	R, monoc.	132 (31.8)	70.3	Suspected	≥60%	Standard Bruce protocol (stepwise increments of 25 Watts every 2 min)	85.1	58.3	80.3
Bokhari S., 2002, USA [39]	R, monoc.	140 (100)	63	Suspected	≥50%	Standard Bruce protocol (stepwise increments of 25 Watts every 2 min)	56	46	51
Rollán M.J., 2002, Spain [40]	P., monoc.	99 (100)	65	Suspected	≥50%	Standard Bruce protocol (stepwise increments of 25 Watts every 2 min)	76	53	66
Cortigiani L., 2003, Italy [41]	P., multic.	71 (21.1)	63	Suspected	≥50%	Standard Bruce protocol (stepwise increments of 25 Watts every 2 min)	82	50	66
Zeng H., 2004, China [42]	P., monoc.	258 (24.8)	59.7	Suspected	≥50%	Standard Bruce protocol (stepwise increments of 25 Watts every 2 min)	77.3	65.9	69.8
González P., 2005, Chile [43]	P., monoc.	145 (33)	60	Known	≥50%	Standard Bruce protocol (stepwise increments of 25 Watts every 2 min)	37	74	43
Nedeljkovic I., 2006, Serbia [44]	P., monoc.	117 (22.2)	54	Suspected	≥50%	Standard Bruce protocol (stepwise increments of 25 Watts every 2 min)	90	87	90
Michaelides A.P., 2007, Greece [45]	P., monoc.	114 (100)	59	Suspected	≥70%	Standard Bruce protocol (stepwise increments of 25 Watts every 2 min)	59	40	50
Bokhari S., 2008, USA [46]	R, monoc.	218 (31)	62	Suspected	≥50%	Standard Bruce protocol (stepwise increments of 25 Watts every 2 min)	36	74	55
Lu C., 2010, Italy [47]	P., monoc.	76 (100)	61	Suspected	≥50%	Modified Bruce protocol (stages “0” and “1/2” performed at 2.7 km/h and 0% and 5% treadmill inclination)	81	56	66
Greulich S., 2012, Germany [48]	P., monoc.	68 (100)	66.4	Suspected	≥70%	Standard Bruce protocol (stepwise increments of 25 Watts every 2 min)	50	73	66
Weustink A.C., 2012, The Netherlands [49]	R, monoc.	376 (32.4)	60.4	Suspected	≥50%	NS	76	47	61.5
Attar A., 2017, Iran [50]	R, multic.	720 (56.5)	53.1	Known	≥50%	NS	98.3	11.9	62.7
*Overall*		*4954* *(51.3)*	*59.8*				*63.6*	*62.3*	*63.2*
**ESE studies**									
Quiñones M.A., 1992, USA [51]	P., monoc.	292 (33.2)	57	Known or suspected	≥50%	Treadmill exercise (stepwise increments of 25 Watts every 2 min)	85	88	86.5
Hecht H.S., 1993, USA [52]	P., monoc.	71 (14)	58	Suspected	≥50%	Supine ergometry on a tilting table (stepwise increments of 25 Watts every 2 min)	90	80	85
Cohen J.L., 1993, USA [53]	P., monoc.	52 (1.9)	63	Suspected	≥70%	Supine ergometry on a tilting table (stepwise increments of 20 Watts every 3 min)	78	87	82.5
Beleslin B.D., 1994, Serbia [54]	P., monoc.	136 (14.7)	50	Known or suspected	≥50%	Treadmill exercise (stepwise increments of 25 Watts every 2 min)	88	82	85
Dagianti A., 1995, Italy [55]	P., monoc.	100 (22)	54	Known or suspected	≥50%	Supine ergometry on a tilting table (stepwise increments of 20 Watts every 3 min)	76	94	85
Bjørnstad K., 1995, Norway [56]	P., monoc.	37 (18.9)	58	Known or suspected	≥50%	Upright bicycle stress test (stepwise increments of 25 Watts every 2 min)	84	67	75.5
Badruddin S.M., 1999, USA [57]	P., monoc.	74 (8.1)	59	Known or suspected	≥50%	Supine ergometry on a tilting table (stepwise increments of 25 Watts every 3 min)	82	80	81
Ha J.W., 2002, USA [58]	R, monoc.	548 (31.4)	65	Suspected	≥50%	Treadmill exercise (stepwise increments of 25 Watts every 2 min)	82	78	80
Shin J.H., 2003, USA [59]	R, monoc.	464 (35)	61	Suspected	≥50%	Treadmill exercise (stepwise increments of 25 Watts every 2 min)	86	87	86.5
Müller H., 2008, Switzerland [60]	P., monoc.	104 (10.6)	61	Known or suspected	≥50%	Supine ergometry on a tilting table (stepwise increments of 20–40 Watts every 2 min)	82	94	88
*Overall*		*1878* *(19.0)*	*58.6*				*83.3*	*83.3*	*83.3*
**Dobutamine echo studies**									
Martin T.W., 1992, USA [61]	P., monoc.	40 (5)	50	Known or suspected	≥50%	Dobutamine IV infusion (3 min dose increments from 10 to 40 mcg/kg/min)	76	60	70
Salustri A., 1992, The Netherlands [26]	P., monoc.	52 (26.9)	58	Known or suspected	≥50%	Dobutamine IV infusion (3 min dose increments from 10 to 40 mcg/kg/min)	62	89	75.5
Marwick T., 1993, Belgium [62]	P., monoc.	217 (28.1)	58	Suspected	≥50%	Dobutamine IV infusion (3 min dose increments from 5 to 40 mcg/kg/min)	72	83	76
Takeuchi M., 1993, Japan [63]	P., monoc.	120 (25.8)	63	Known or suspected	≥50%	Dobutamine IV infusion (5 min dose increments from 5 to 30 mcg/kg/min)	85	93	88
Cohen J.L., 1993, USA [53]	P., monoc.	52 (1.9)	63	Suspected	≥70%	Dobutamine IV infusion (3 min dose increments from 2.5 to 40 mcg/kg/min)	86	87	87
Beleslin B.D., 1994, Serbia [54]	P., monoc.	136 (14.7)	50	Known or suspected	≥50%	Dobutamine IV infusion (3 min dose increments from 5 to 40 mcg/kg/min)	82	77	82
Panza J.A., 1994, USA [28]	P., monoc.	76 (18.4)	60	Known or suspected	≥70%	Dobutamine IV infusion (5 min dose increments from 2.5 to 40 mcg/kg/min)	89	100	91
Dagianti A., 1995, Italy [55]	P., monoc.	100 (22)	54	Known or suspected	≥70%	Dobutamine IV infusion (5 min dose increments from 5 to 40 mcg/kg/min)	72	97	87
Ho F.M., 1995, Taiwan [64]	P., monoc.	54 (14.8)	58	Known or suspected	≥50%	Dobutamine IV infusion (3 min dose increments from 5 to 40 mcg/kg/min)	93	73	89
San Román J.A., 1996, Spain [29]	P., monoc.	102 (44.1)	62	Suspected	≥50%	Dobutamine IV infusion (3 min dose increments from 10 to 40 mcg/kg/min plus atropine 0.25–1 mg)	77	95	95
Pingitore A., 1996, Italy [65]	P., monoc.	360 (16.6)	60	Known or suspected	≥50%	Dobutamine IV infusion (3 min dose increments from 5 to 40 mcg/kg/min plus atropine 0.25–1 mg)	84	89	89
Kisacik H.L.,1996, Turkey [30]	P., monoc.	69 (15.9)	51	Known or suspected	≥50%	Dobutamine IV infusion (3 min dose increments from 5 to 40 mcg/kg/min plus atropine 0.25–1 mg)	94	86	91
Hennessy T.G., 1997, Ireland [33]	P., monoc.	116 (29.3)	59	Suspected	≥50%	Dobutamine IV infusion (3 min dose increments from 10 to 40 mcg/kg/min plus atropine 0.25–1 mg)	82	63	72.5
Santoro G.M., 1998, Italy [36]	P., monoc.	60 (NS)	NS	Suspected	≥70%	Dobutamine IV infusion (3 min dose increments from 10 to 40 mcg/kg/min plus atropine 0.25–1 mg)	61	96	77
San Román J.A., 1998, Spain [37]	P., monoc.	102 (51)	64	Suspected	≥50%	Dobutamine IV infusion (3 min dose increments from 10 to 40 mcg/kg/min plus atropine 0.25–1 mg)	78	88	82
Elhendy A., 1998, The Netherlands [66]	P., monoc.	84 (36.9)	60	Known or suspected	≥50%	Dobutamine IV infusion (3 min dose increments from 5 to 40 mcg/kg/min plus atropine 0.25–1 mg)	73	83	75
Fragasso G., 1999, Italy [67]	P., monoc.	101 (45.5)	61	Suspected	≥50%	Dobutamine IV infusion (3 min dose increments from 5 to 40 mcg/kg/min)	88	80	84
Previtali M., 1999, Italy [68]	P., monoc.	43 (2.3)	53	Known	≥50%	Dobutamine IV infusion (3 min dose increments from 5 to 40 mcg/kg/min plus atropine 0.25–1 mg)	79	60	77
Ciaroni S., 2000, Switzerland [69]	P., monoc.	29 (31)	71	Suspected	≥50%	Dobutamine IV infusion (3 min dose increments from 5 to 40 mcg/kg/min plus atropine 0.25–1 mg)	88	92	90
Smart S.C., 2000, USA [70]	P., multic.	183 (27.3)	60	Known or suspected	≥50%	Dobutamine IV infusion (5 min dose increments from 10 to 40 mcg/kg/min plus atropine 0.25–1 mg)	87	91	89
Geleijnse M.L., 2000, The Netherlands [71]	P., monoc.	64 (62.5)	59	Suspected	≥50%	Dobutamine IV infusion (3 min dose increments from 10 to 40 mcg/kg/min plus atropine 0.25–1 mg)	68	91	84
Elhendy A., 2000, The Netherlands [72]	P., monoc.	91 (49.4)	57	Known	≥50%	Dobutamine IV infusion (3 min dose increments from 5 to 40 mcg/kg/min plus atropine 0.25–1 mg)	56	84	67
Tandoğan I., 2001, Turkey [73]	P., monoc.	26 (30.8)	57	Suspected	≥50%	Dobutamine IV infusion (3 min dose increments from 5 to 40 mcg/kg/min plus atropine 0.25–1 mg)	91	92	92
Lancellotti P., 2001, Belgium [74]	P., monoc.	75 (18.7)	56	Known	≥50%	Dobutamine IV infusion (3 min dose increments from 5 to 40 mcg/kg/min plus atropine 0.25–1 mg)	78	83	79
Nedeljkovic I., 2006, Serbia [44]	P., monoc.	117 (22.2)	54	Suspected	≥50%	Dobutamine IV infusion (3 min dose increments from 5 to 40 mcg/kg/min plus atropine 0.25–1 mg)	96	92	94
*Overall*		*2469* *(26.9)*		*58.4*			*79.2*	*84.7*	*82.9*
**Dipyridamole echo studies**									
Martin T.W., 1992, USA [61]	P., monoc.	40 (5)	50	Known or suspected	≥50%	Dipyridamole IV infusion (2-step: 0.56–0.84 mg/kg over 10 min)	56	67	61.5
Beleslin B.D., 1994, Serbia [54]	P., monoc.	136 (14.7)	50	Known or suspected	≥50%	Dipyridamole IV infusion (2-step: 0.56–0.84 mg/kg over 10 min)	74	94	84
Dagianti A., 1995, Italy [55]	P., monoc.	100 (22)	54	Known or suspected	≥50%	Dipyridamole IV infusion (2-step: 0.56–0.84 mg/kg over 10 min)	52	97	74.5
Bjørnstad K., 1995, Norway [56]	P., monoc.	37 (18.9)	58	Known or suspected	≥50%	Dipyridamole IV infusion (2-step: 0.56–0.84 mg/kg over 10 min)	68	100	84
San Román J.A., 1996, Spain [29]	P., monoc.	102 (44.1)	6	Suspected	≥50%	Dipyridamole IV infusion (0.84 mg/kg over 6 min)	77	97	87
Pingitore A., 1996, Italy [65]	P., monoc.	360 (16.6)	60	Known or suspected	≥50%	Dipyridamole IV infusion (2-step: 0.56–0.84 mg/kg over 10 min plus atropine 0.25–1 mg)	82	94	88
Santoro G.M., 1998, Italy [36]	P., monoc.	60 (NS)	NS	Suspected	≥70%	Dipyridamole IV infusion (2-step: 0.56–0.84 mg/kg over 10 min)	55	96	75.5
San Román J.A., 1998, Spain [37]	P., monoc.	102 (51)	64	Suspected	≥50%	Dipyridamole IV infusion (0.84 mg/kg over 6 min)	81	90	85.5
Fragasso G., 1999, Italy [67]	P., monoc.	101 (45.5)	61	Suspected	≥50%	Dipyridamole IV infusion (2-step: 0.56–0.84 mg/kg over 6 min)	61	91	76
Vigna C., 2001, Italy [75]	P., monoc.	54 (48.1)	59	Suspected	≥50%	Dipyridamole IV infusion (2-step: 0.56–0.84 mg/kg over 10 min)	70.6	94.6	82.6
Cortigiani L., 2003, Italy [41]	P., multic.	71 (21.1)	63	Suspected	≥70%	Dipyridamole IV infusion (2-step: 0.56–0.84 mg/kg over 10 min plus atropine 0.25–1 mg)	82	89	85.5
Vigna C., 2006, Italy [76]	P., monoc.	27 (8)	63	Suspected	≥70%	Dipyridamole IV infusion (2-step: 0.56–0.84 mg/kg over 10 min)	42	93	67.5
Nedeljkovic I., 2006, Serbia [44]	P., monoc.	117 (22.2)	54	Suspected	≥50%	Dipyridamole IV infusion (2-step: 0.56–0.84 mg/kg over 10 min plus atropine 0.25–1 mg)	93	92	92.5
*Overall*		*1307* *(26.4)*	*53.5*				*68.7*	*91.9*	*80.3*
**Dual imaging echo studies**									
Rigo F., 2003, Italy [77]	P., monoc.	230 (41.7)	63.5	Suspected	≥50%	Dipyridamole IV infusion (2-step: 0.56–0.84 mg/kg over 10 min) Rest and peak stress CFR	93	80.6	86.8
Lowenstein J., 2003, Argentina [78]	P., monoc.	752 (36.4)	64.7	Suspected	≥70%	Dipyridamole IV infusion (0.84 mg/kg over 4 min plus atropine 1 mg) Rest and peak stress CFR	86.8	73.2	80
Nohtomi Y., 2003, Japan [79]	P., monoc.	110 (28.2)	65	Known or suspected	≥50%	Dipyridamole IV infusion (0.84 mg/kg over 6 min plus atropine 0.25–1 mg) Rest and peak stress CFR	94	65	79.5
Ascione L., 2006, Italy [80]	P., monoc.	159 (31)	59	Known	≥70%	Dipyridamole IV infusion (0.84 mg/kg over 6 min) Rest and peak stress CFR	85	87	86
Gaibazzi N., 2010, Italy [81]	P., multic.	400 (34.2)	66	Known or suspected	≥50%	Dipyridamole IV infusion (0.84 mg/kg over 6 min) Rest and peak stress CFR	84	71	80
Cortigiani L., 2011, Italy* [82]	P., multic.	1411 (39)	66	Known or suspected	≥75%	Dipyridamole IV infusion (0.84 mg/kg over 6 min) Rest and peak stress CFR	87	76	81.5
Cortigiani L., 2011, Italy** [82]	P., multic.	678 (35)	60	Known or suspected	≥75%	Dipyridamole IV infusion (0.84 mg/kg over 6 min) Rest and peak stress CFR	89	80	84.5
Kasprzak J.D., 2013, Poland [83]	P., monoc.	64 (31.2)	58	Known or suspected	≥50%	Dipyridamole IV infusion (0.84 mg/kg over 4 min plus atropine 0.25–1 mg) Rest and peak stress CFR	68	84	76
Pichel I.Á., 2019, Spain [84]	P., monoc.	74 (28)	60.3	Known or suspected	≥50%	Dipyridamole IV infusion (0.84 mg/kg over 6 min) Rest and peak stress CFR	72.7	49.2	61
*Overall*		*3878* *(33.9)*	*62.5*				*84.4*	*74.0*	*79.5*
**Exercise SPECT studies**									
Stewart R.E., 1991, USA [85]	P., monoc.	81 (35.8)	57	Known or suspected	≥50%	Tl–201 SPECT (standard Bruce protocol)	84	53	79
Prisant L.M., 1992, USA [86]	P., monoc.	92 (46)	54.8	Suspected	≥50%	Tl–201 SPECT (standard Bruce protocol)	94.4	63.5	69.6
Gupta N.C., 1992, USA [87]	P., multic.	93 (17.2)	57.9	Suspected	≥50%	Tl–201 SPECT (standard Bruce protocol)	81.8	80	81.2
Quiñones M.A., 1992, USA [51]	P., monoc.	292 (33.2)	57	Known or suspected	≥50%	Tl–201 SPECT (standard Bruce protocol)	85	81	83
Hecht H.S., 1993, USA [52]	P., monoc.	71 (14)	58	Known or suspected	≥50%	Tl–201 SPECT (standard Bruce protocol)	92	65	85
Zammarchi A., 1994, Italy [88]	P., monoc.	54 (37)	60.8	Suspected	≥70%	Tl–201 SPECT (standard Bruce protocol)	50	47	48
Fleming R.M., 1995, USA [89]	P., monoc.	159 (38.9)	62.9	Suspected	≥50%	Tl–201 SPECT (standard Bruce protocol)	92.5	42.8	67.6
Fragasso G., 1999, Italy [67]	P., monoc.	101 (45.5)	61	Suspected	≥50%	Tc–99m sestamibi SPECT (standard Bruce protocol)	98	36	71
Previtali M., 1999, Italy [68]	P., monoc.	43 (2.3)	53	Known	≥50%	Tl–201 SPECT (standard Bruce protocol)	76	60	74
Ciaroni S., 2000, Switzerland [69]	P., monoc.	29 (31)	71	Suspected	≥50%	Tl–201 SPECT (standard Bruce protocol)	94	31	62.5
Doğruca Z., 2000, Turkey [90]	P., monoc.	38 (10.5)	58.2	Known or suspected	≥50%	Tl–201 prone SPECT and Tc–99 m sestamibi SPECT (standard Bruce protocol)	90	54	78
Tandoğan I., 2001, Turkey [73]	P., monoc.	26 (30.8)	57	Suspected	≥50%	Tl–201 SPECT (standard Bruce protocol)	100	42	69
Gentile R., 2001, Italy [38]	R, monoc.	132 (31.8)	70.3	Suspected	≥60%	Tl–201 SPECT (standard Bruce protocol)	93.5	54.1	86.3
González P., 2005, Chile [43]	P., monoc.	145 (33)	60	Known or suspected	≥50%	Tl–201 SPECT (standard Bruce protocol)	87	57	81
Bokhari S., 2008, USA [46]	R, monoc.	218 (31)	62	Suspected	≥50%	Tl–201 SPECT (standard Bruce protocol)	81	79	80
Weustink A.C., 2012, The Netherlands [49]	R, monoc.	376 (32.4)	60.4	Suspected	≥50%	Tc–99m sestamibi SPECT (standard Bruce protocol)	89	77	83
Raman S.V., 2016, USA [91]	P., multic.	94 (46)	57.1	Known or suspected	≥70%	Tc–99m sestamibi SPECT (standard Bruce protocol)	50	93.7	71.8
Ahmad I.G., 2016, USA [92]	P., monoc.	85 (31)	56.3	Suspected	≥50%	Tl–201 SPECT * and Tc–99m sestamibi SPECT ** (standard Bruce protocol)	84	91	88
*Overall*		*2129* *(30.4)*	*59.7*				*84.6*	*61.5*	*75.4*
**Dobutamine SPECT studies**									
Marwick T., 1993, Belgium [62]	P., monoc.	217 (28.1)	58	Suspected	≥50%	Tc–99m sestamibi SPECT (dobutamine IV infusion: 3 min dose increments from 5 to 40 mcg/kg/min)	76	67	73
Fleming R.M., 1995, USA [89]	P., monoc.	159 (38.9)	62.9	Suspected	≥50%	Tl–201 SPECT, teboroxime SPECT, and Tc–99m sestamibi SPECT (dobutamine IV infusion: 3 min dose increments from 5 to 40 mcg/kg/min)	100	100	100
Kisacik H.L.,1996, Turkey [30]	P., monoc.	69 (15.9)	51	Known or suspected	≥50%	Tc–99m sestamibi SPECT (dobutamine IV infusion: 3 min dose increments from 5 to 40 mcg/kg/min)	96	64	84
Santoro G.M., 1998, Italy [36]	P., monoc.	60 (NS)	NS	Suspected	≥70%	Tc–99m sestamibi SPECT (dobutamine IV infusion: 3 min dose increments from 10 to 40 mcg/kg/min plus atropine 0.25–1 mg)	91	81	87
San Román J.A., 1998, Spain [37]	P., monoc.	102 (51)	64	Suspected	≥50%	Tc–99m sestamibi SPECT (dobutamine IV infusion: (3 min dose increments from 10 to 40 mcg/kg/min plus atropine 0.25–1 mg)	87	70	81
Elhendy A., 1998, The Netherlands [66]	P., monoc.	84 (36.9)	60	Suspected	≥50%	Tc–99m sestamibi SPECT (dobutamine IV infusion: 3 min dose increments from 5 to 40 mcg/kg/min plus atropine 0.25–1 mg)	67	83	70
Elhendy A., 2000, The Netherlands [72]	P., monoc.	91 (49.4)	57	Suspected	≥50%	Tc–99m sestamibi SPECT (dobutamine IV infusion: 3 min dose increments from 5 to 40 mcg/kg/min plus atropine 0.25–1 mg)	56	73	63
Lancellotti P., 2001, Belgium [74]	P., monoc.	75 (18.7)	56	Known	≥50%	Tc–99m sestamibi SPECT (dobutamine IV infusion: 3 min dose increments from 5 to 40 mcg/kg/min plus atropine 0.25–1 mg)	70	83	71
Olszowska M., 2003, Poland [93]	P., monoc.	44 (45.4)	58.9	Suspected	≥60%	Tc–99m sestamibi SPECT (dobutamine IV infusion: 3 min dose increments from 5 to 40 mcg/kg/min)	93	84	86
*Overall*		*901* *(35.5)*	*58.5*				*81.8*	*78.3*	*79.4*
**Dipyridamole SPECT studies**									
Takeuchi M., 1993, Japan [63]	P., monoc.	120 (25.8)	63	Known or suspected	≥50%	Tl–201 SPECT (dipyridamole IV infusion: 0.56 mg/kg over 4 min)	89	85	88
Ho F.M., 1995, Taiwan [64]	P., monoc.	54 (14.8)	58	Known or suspected	≥50%	Tl–201 SPECT (dipyridamole IV infusion: 0.56 mg/kg over 4 min)	98	73	93
Fleming R.M., 1995, USA [89]	P., monoc.	159 (38.9)	62.9	Suspected	≥50%	Tl–201 SPECT, teboroxime SPECT, and Tc–99m sestamibi SPECT (dipyridamole IV infusion: 0.85 mg/kg over 4 min)	100	88.9	94.4
Cramer M.J., 1996, The Netherlands [94]	P., monoc.	39 (28.2)	63	Known or suspected	≥50%	Tc–99m sestamibi SPECT (dipyridamole IV infusion: 0.84 mg/kg over 10 min)	93	100	96.5
Santoro G.M., 1998, Italy [36]	P., monoc.	60 (NS)	NS	Suspected	≥70%	Tc–99m sestamibi SPECT (dipyridamole IV infusion: 0.84 mg/kg over 10 min)	97	89	93
Smart S.C., 2000, USA [70]	P., multic.	183 (27.3)	60	Known or suspected	≥50%	Tc–99m sestamibi SPECT (dipyridamole IV infusion: 0.84 mg/kg over 4 min)	80	73	76.5
Senior R., 2004, United Kingdom [95]	P., multic.	55 (18)	61	Suspected	≥50%	Tc–99m tetrofosmin SPECT (dipyridamole IV infusion: 0.84 mg/kg over 6 min)	49	92	70.5
Jeetley P., 2006, United Kingdom [96]	P., multic.	123 (29)	62	Known or suspected	≥50%	Tc–99m sestamibi SPECT (dipyridamole IV infusion: 0.84 mg/kg over 6 min)	82	52	67
Vigna C., 2006, Italy [76]	P., monoc.	27 (8)	63	Suspected	≥70%	Tc–99m sestamibi SPECT (dipyridamole IV infusion: 0.84 mg/kg over 10 min)	67	53	60
*Overall*		*820* *(23.8)*	*61.6*				*83.9*	*78.4*	*82.1*
**Adenosine SPECT studies**									
LaManna M.M., 1990, USA [97]	P., multic.	15 (NS)	58	Known	≥50%	Tl–201 SPECT (adenosine IV infusion: 140 mcg/kg/min over 6 min)	77	100	88.5
Gupta N.C., 1992, USA [87]	P., multic.	93 (17.2)	57.9	Suspected	≥50%	Tl–201 SPECT (adenosine IV infusion: 140 mcg/kg/min over 6 min)	83.3	86.6	84.3
Cramer M.J., 1996, The Netherlands [94]	P., monoc.	39 (28.2)	63	Suspected	≥50%	Tc–99m sestamibi SPECT (adenosine IV infusion: 140 mcg/kg/min over 6 min)	90	100	95
Karavidas A.I., 2006, Greece [98]	P., monoc.	47 (38.3)	55	Suspected	≥50%	Tl–201 SPECT (adenosine IV infusion: 140 mcg/kg/min over 6 min)	73	72	72
Aggeli C., 2007, Greece [99]	P., monoc.	50 (32)	67	Suspected	≥50%	Tl–201 SPECT (adenosine IV infusion: 140 mcg/kg/min over 6 min)	80	94	85
Futamatsu H., 2008, USA [100]	P., monoc.	24 (50)	60	Suspected	≥50%	Tc–99m sestamibi SPECT (adenosine IV infusion: 140 mcg/kg/min over 6 min)	67.4	81.3	74.3
Greenwood J.P., 2014, United Kingdom [101]	P., monoc.	628 (37.4)	60	Suspected	≥70%	Tc–99m tetrofosmin SPECT (adenosine IV infusion: 140 mcg/kg/min over 4 min)	70.8	81.3	76
*Overall*		*896* *(33.9)*	*60.1*				*77.4*	*87.9*	*82.2*
**Exercise CMR studies**									
Rerkpattanapipat P., 2003, USA [102]	P., monoc.	27 (26)	62	Suspected	70	Cine CMR at 1.5 Tesla (treadmill exercise test: standard Bruce protocol)	79	85	82
Raman S.V., 2016, USA [91]	P., multic.	94 (46)	57.1	Known or suspected	70	Cine CMR at 1.5 Tesla (treadmill exercise test: standard Bruce protocol)	78.6	98.7	88.6
Ochs A., 2025, Germany [103]	P., monoc.	260 (25)	64	Known or suspected	75	Cine CMR at 1.5 or 3 Tesla (dynamic handgrip exercise)	53	93	84
*Overall*		*381* *(32.3)*	*61*				*70.2*	*92.2*	*84.9*
**Dobutamine CMR studies**									
van Rugge F.P., 1993, The Netherlands [27]	P., monoc.	45 (20)	61	Suspected	50	Cine CMR at 1.5 Tesla (dobutamine IV infusion up to 20 mcg/kg/min)	81	100	84
Nagel E., 1999, Germany [104]	P., monoc.	208 (29.3)	60	Suspected	50	Cine CMR at 1.5 Tesla (dobutamine IV infusion up to 40 mcg/kg/min plus atropine 0.25–1 mg)	86.2	85.7	85.9
al–Saadi N., 2002, Germany [105]	P., monoc.	27 (25.9)	56	Known or suspected	75	Cine CMR at 1.5 Tesla (dobutamine IV infusion up to 20 mcg/kg/min)	81	73	77
Paetsch I., 2004, Germany [106]	P., multic.	79 (34.2)	61	Known or suspected	50	Cine CMR at 1.5 Tesla (dobutamine IV infusion up to 40 mcg/kg/min plus atropine up to 2 mg)	89	80	86
Wahl A., 2004, Germany [107]	P., multic.	160 (18)	59	Known or suspected	50	Cine CMR at 1.5 Tesla (dobutamine IV infusion up to 40 mcg/kg/min plus atropine up to 2 mg)	89	84	88
Kelle S., 2008, Germany [108]	P., monoc.	30 (20)	66	Known or suspected	50	Cine CMR at 1.5 or 3 Tesla (dobutamine IV infusion up to 40 mcg/kg/min plus atropine up to 2 mg)	80	85.7	81.8
Heilmaier C., 2009, Germany [109]	P., monoc.	50 (22)	62	Known or suspected	50	Cine CMR at 1.5 Tesla (dobutamine IV infusion up to 40 mcg/kg/min plus atropine up to 1 mg)	86	90	89
Gebker R., 2010, Germany [110]	P., monoc.	745 (27.4)	63.5	Known or suspected	70	Cine CMR at 1.5 Tesla (dobutamine IV infusion up to 40 mcg/kg/min plus atropine up to 2 mg)	86	84	85
Mordi I., 2014, United Kingdom [111]	P., monoc.	82 (35.4)	56.5	Suspected	70	Cine CMR at 1.5 Tesla (dobutamine IV infusion up to 40 mcg/kg/min plus atropine up to 2 mg)	82.4	95.8	90.2
Weberling L.D., 2023, Germany [112]	P., monoc.	176 (27.8)	60.9	Suspected	70	Cine CMR at 1.5 or 3 Tesla (dobutamine IV infusion up to 40 mcg/kg/min plus atropine up to 2 mg)	71.4	98.4	85
*Overall*		*1602* *(26.0)*	*60.6*				*83.2*	*87.7*	*85.2*
**Dipyridamole CMR studies**									
Baer F.M., 1993, Germany [113]	P., monoc.	33 (3)	58	Known or suspected	70	Cine CMR at 1.5 Tesla (dipyridamole IV infusion: 0.75 mg/kg over 10 min)	84	89	86.5
Hartnell G., 1994, USA [114]	P., monoc.	18 (22.2)	57	Known or suspected	70	Cine CMR at 1 Tesla (dipyridamole IV infusion: 0.56 mg/kg over 4 min)	83	100	91.5
Zhao S., 1997, France [115]	P., monoc.	16 (14.3)	60	Known or suspected	70	Cine CMR at 1.5 Tesla (dipyridamole IV infusion: 0.56 mg/kg over 4 min)	80	75	77.5
Takase B., 2004, Japan [116]	P., monoc.	102 (21.6)	66	Known or suspected	50	Cine CMR at 1.5 Tesla (dipyridamole IV infusion: 0.56 mg/kg over 4 min)	93	85	89
Stauder N.I., 2007, Switzerland [117]	P., monoc.	45 (13.3)	63.8	Known or suspected	50	Cine CMR at 1.5 Tesla (dipyridamole IV infusion: 0.56 mg/kg over 4 min)	95.2	96.8	96
Pingitore A., 2008, Italy [118]	P., monoc.	93 (34.4)	64	Known or suspected	50	Cine CMR at 1.5 Tesla (dipyridamole IV infusion: 0.84 mg/kg over 6 min)	82	96	86
Mordini F.E., 2014, USA [119]	P., monoc.	67 (33)	60	Suspected	70	Cine CMR at 1.5 Tesla (dipyridamole IV infusion: 0.56 mg/kg over 4 min)	87	93	90
Deva D.P., 2014, Canada [120]	R, monoc.	19 (41)	32.4	Suspected	NS	Cine CMR at 1.5 or 3 Tesla (dipyridamole IV infusion: 0.56 mg/kg over 4 min)	82	100	91
Yun C.H., 2015, Taiwan [121]	P., monoc	58 (29.3)	59	Known or suspected	70	Cine CMR at 3 Tesla (dipyridamole IV infusion: 0.56 mg/kg over 4 min)	77	80	78.5
*Overall*		*451* *(23.6)*	*57.8*				*84.8*	*90.5*	*87.3*
**Adenosine CMR studies**									
Paetsch I., 2004, Germany [106]	P., multic.	79 (34.2)	61	Known or suspected	50	Cine CMR at 1.5 Tesla (adenosine IV infusion: 140 mcg/kg/min for 6 min)	40	96	68
Klem I., 2006, USA [122]	P., monoc.	92 (51)	58	Suspected	70	Cine CMR at 1.5 Tesla (adenosine IV infusion: 140 mcg/kg/min for 2 min)	84	58	71
Klein C., 2008, Germany [123]	P., monoc.	54 (35.2)	60	Suspected	50	Cine CMR at 1.5 Tesla (adenosine IV infusion: 140 mcg/kg/min for 4 min)	87	88	87.5
Futamatsu H., 2008, USA [100]	P., monoc.	24 (50)	60	Suspected	50	Cine CMR at 1.5 Tesla (adenosine IV infusion: 140 mcg/kg/min for 4 min)	74.4	79.4	76.9
Arnold J.R., 2010, United Kingdom [124]	P., monoc.	65 (35)	64	Suspected	50	Cine CMR at 3 Tesla (adenosine IV infusion: 140 mcg/kg/min for 4 min)	90	81	85.5
Bettencourt N., 2013, Portugal [125]	P., monoc.	43 (35)	61	Suspected	50	Cine CMR at 1.5 Tesla (adenosine IV infusion: 140 mcg/kg/min for 4 min)	79	95	87
Salerno M., 2014, USA [126]	P., monoc.	41 (32)	62	Known or suspected	50	Cine CMR at 1.5 Tesla (adenosine IV infusion: 140 mcg/kg/min for 3 min)	89	85	87
Greenwood J.P., 2014, United Kingdom [101]	P., monoc.	628 (37.4)	60	Suspected	70	Cine CMR at 1.5 Tesla (adenosine IV infusion: 140 mcg/kg/min for 4 min)	85.6	82.8	84.2
Manka R., 2015, Switzerland [127]	P., multic.	150 (30)	62.9	Suspected	50	Cine CMR at 1.5 Tesla (adenosine IV infusion: 140 mcg/kg/min for 6 min)	84.7	90.8	87.75
Ahmad I.G., 2016, USA [92]	P., monoc.	85 (31)	56.3	Suspected	50	Cine CMR at 1.5 Tesla (adenosine IV infusion: 140 mcg/kg/min for 2 min)	85	93	89
Ntsinjana H.N., 2017, United Kingdom [128]	P., monoc.	58 (31)	14.1	Suspected	50	Cine CMR at 1.5 Tesla (adenosine IV infusion: 140 mcg/kg/min for 4 min)	100	98	99
Foley J.R.J., 2017, United Kingdom [129]	P., monoc.	27 (15)	65	Known or suspected	70	Cine CMR at 1.5 Tesla (adenosine IV infusion: 140 mcg/kg/min for 4 min)	81	96	88.5
*Overall*		*1346* *(34.7)*	*57*				*81.6*	*86.9*	*84.3*

**Table 2:** List of the principal factors that may affect sensitivity and specificity of EST in the detection of obstructive CAD. CAD, coronary artery disease; LBBB, left bundle branch block; ECG, electrocardiogram; EST, exercise stress testing; LVH, left ventricular hypertrophy; and WPW, Wolff–Parkinson–White.

Factors Reducing EST Sensitivity	Factors Reducing EST Specificity
Inadequate myocardial stress (e.g., submaximal exertion)	Hypertensive response to exercise, LVH
Lower exercise capacity (e.g., commonly in females)	Female sex
Medications reducing heart rate response (β-blockers, nitrates, and calcium channel blockers)	Estrogen replacement therapy
Insufficient ECG lead monitoring	Severe aortic stenosis, severe aortic or mitral regurgitation, and mitral valve prolapse
Caffeine intake before test	Cardiomyopathies
Single-vessel disease	Anemia and hypokalemia
Right coronary or circumflex artery disease	Baseline ECG abnormalities (e.g., LBBB, paced rhythm, and WPW), digitalis use
Serial stenoses or extensive collateral circulation	Heightened myocardial sensitivity to catecholamines
Posterior wall ischemia (not reflected on standard ECG leads)	Rapid ST segment recovery post-exercise
Balanced underperfusion in multivessel disease	Chest wall deformities (e.g., concave-shaped chest)

**Table 3:** Strengths and weaknesses of the four stress echocardiographic approaches in clinical practice. CAD, coronary artery disease; CFR, coronary flow reserve; DipSE, dipyridamole stress echocardiography; LAD, left anterior descending artery; and WM, wall motion.

Technique	Main Advantages	Main Limitations
**Exercise Stress Echocardiography**	Physiological stress; high specificity; good vessel localization; superior detection in LAD and multivessel disease; inexpensive and widely available.	Operator-dependent; technically challenging during exercise; limited in hypertensive, elderly, or deconditioned patients; false positives in women and hypertensive response.
**Dobutamine Stress Echocardiography**	High sensitivity in one- and multivessel disease; useful for patients unable to exercise; fewer artifacts; enhanced by atropine; suitable for perioperative risk assessment.	Lower specificity than DipSE; may induce arrhythmias; false positives in cardiomyopathy; reduced accuracy in mild disease; may require atropine with beta-blockers.
**Dipyridamole Stress Echocardiography**	Very high specificity; well tolerated; suitable for patients unable to exercise; low arrhythmia risk; effective with atropine coadministration.	Lower sensitivity for single-vessel or mild disease; affected by antianginal therapy; may not provoke ischemia in moderate CAD; requires atropine to enhance sensitivity.
**Dual-imaging Stress Echocardiography (WM + CFR)**	Improved sensitivity over wall motion alone; effective in LAD detection; helpful in early or microvascular disease; good risk stratification; no radiation.	Technically demanding; limited to LAD; requires expertise; cannot distinguish micro- vs. macrovascular disease; time-consuming.

**Table 4:** Comparison of stress modalities in myocardial SPECT imaging. AV, atrioventricular; BP, blood pressure; CAD, coronary artery disease; ECG, electrocardiogram; EST, exercise stress testing; LAD, left anterior descending artery; LBBB, left bundle branch block; LVH, left ventricular hypertrophy; and SPECT, single-photon emission computed tomography.

Stress Modality	Advantages	Limitations
**Exercise Stress SPECT**	• Physiological stress with prognostic value • Superior to EST in detecting CAD (especially in single-vessel disease) • Useful for assessing functional capacity • Early detection of perfusion defects	• Not feasible in 25–30% of patients • High false-positive rate in women (breast attenuation) and patients with LBBB • Attenuation artifacts in inferior wall • Requires ability to exercise
**Dobutamine Stress SPECT**	• Suitable for patients unable to exercise • High sensitivity and specificity in LAD and single-vessel disease • Preferred in patients with LBBB or LVH • Comparable to stress echocardiography	• Risk of arrhythmias and BP fluctuations • Interference with MIBI uptake • Less sensitive for mild CAD • Operator dependent
**Dipyridamole Stress SPECT**	• Effective vasodilator for non-exercise testing • High sensitivity in detecting CAD and multivessel disease • Better for identifying mild-to-moderate stenosis • Generally well tolerated; reversible side effects	• Lower specificity due to attenuation artifacts • Contraindicated in bronchospastic disease • Risk of motion-related false positives • Requires aminophylline for reversal of side effects
**Adenosine Stress SPECT**	• Rapid onset and short half-life • Excellent concordance with exercise SPECT • Superior in patients with LBBB • Well tolerated with short infusion time	• Can cause chest pain, AV block, or bradycardia • Less accurate in single-vessel disease • Requires continuous ECG and BP monitoring • Reduced specificity in small hearts

**Table 5:** Principal advantages and limitations of exercise stress CMR, dobutamine stress CMR, dipyridamole, stress CMR, and adenosine stress CMR in clinical practice. CAD, coronary artery disease; CKD, chronic kidney disease; DSE, dobutamine stress echocardiography; DSCMR, dobutamine stress cardiac magnetic resonance; IV, intravenous; MR, magnetic resonance; and SPECT, single-photon emission computed tomography.

Stress CMR Method	Advantages	Limitations
**Exercise Stress CMR**	Physiological stress; no pharmacologic agents; detects epicardial and microvascular disease; superior to SPECT.	Requires fast post-exercise imaging; logistical challenges; motion artifacts; limited adoption.
**Dobutamine Stress CMR**	High spatial/temporal resolution; superior to DSE; rapid onset/offset; good for multivessel CAD.	Contraindicated in arrhythmia; ECG changes hard to assess; MR-incompatibility issues; breath-holding needed; suboptimal temporal resolution at high heart rates.
**Dipyridamole Stress CMR**	Effective for perfusion and wall motion; high diagnostic accuracy; safe in CKD; good for moderate stenoses.	Longer protocol; contraindicated in asthma; not widely available; lower specificity than DSCMR in severe CAD.
**Adenosine Stress CMR**	Short test time; high spatial resolution; well tolerated; excellent accuracy; useful in pediatrics and women.	Requires IV access; contraindications include asthma; may produce artifacts; does not test exercise capacity.

**Table 6:** ESC 2024 guideline recommendations for the clinical application of EST, SE, stress myocardial SPECT, and stress CMR. BP, blood pressure; CACS, coronary artery calcium score; CAD, coronary artery disease; CFR, coronary flow reserve; CMR, cardiac magnetic resonance; EST, exercise stress testing; MACE, major adverse cardiovascular events; PET, positron emission tomography; SE, stress echocardiography; and SPECT, single-photon emission computed tomography.

Test	Clinical Use	Recommendations (ESC 2024) [6]
**EST**	Low pre-test likelihood (5–15%) of CAD; assessment of symptoms, arrhythmias, and BP response.	May be used to reclassify patients to very low risk (≤5%) when negative. Recommended for functional capacity and symptom evaluation (Class I C). May be used when imaging is unavailable (Class IIb B).
**SE**	Moderate to high pre-test likelihood (15–85%) of CAD; evaluation of myocardial ischemia, arrhythmias, and microvascular dysfunction.	Recommended to diagnose ischemia and estimate MACE risk (Class I B). Doppler CFR and myocardial contrast agents may be used to enhance diagnostic and prognostic value (Class IIb B and Class I B).
**Stress myocardial SPECT**	Moderate to high pre-test likelihood (15–85%) of CAD; diagnosis and quantification of ischemia and/or scarring.	Recommended for diagnosis and risk assessment in moderate-to-high risk patients (Class I B). CACS should be measured when SPECT/PET is used (Class I B). Prefer PET when available
**Stress CMR**	Moderate to high pre-test likelihood (15–85%) of CAD; comprehensive assessment of perfusion, function, and viability.	Recommended for diagnosis of ischemia and scarring, and risk stratification (Class I B). Use in cases needing additional information beyond perfusion.

## Data Availability

Data extracted from included studies will be publicly available on Zenodo (https://zenodo.org, accessed on 1 August 2025).

## Supplementary Materials

The following supporting information can be downloaded at https://www.mdpi.com/article/10.3390/jcm14176238/s1: Table S1: RoB assessment of the included studies. Tables S2–S4: subgroup analyses by region, period, and design, stratified by modality.
