# Supplementary material for: Diagnostic Accuracy of Exercise Stress Testing, Stress Echocardiography, Myocardial Scintigraphy, and Cardiac Magnetic Resonance for Obstructive Coronary Artery Disease: Systematic Reviews and Meta-Analyses of 104 Studies Published from 1990 to 2025
Source: J Clin Med. 2025 Sep 4;14(17):6238. doi: 10.3390/jcm14176238 (PMC12429821; doi:10.3390/jcm14176238)
Supplement: Supplementary file 1 [file jcm-14-06238-s001.zip › jcm-3840284-supplementary.pdf]

Table S1. RoB assessment of the included studies.

NIH Quality Assessment Tool for Observational Cohort and Cross-Sectional Studies															
**Study name**	**Method/s**	**Q1**	**Q2**	**Q3**	**Q4**	**Q5**	**Q6**	**Q7**	**Q8**	**Q9**	**Q10**	**Q11**	**Q12**	**Q13**	**Q14**	**Quality**
LaManna M.M., 1990	AdenoSPECT	Yes	Yes	Yes	Yes	No	Yes	Yes	No	Yes	No	Yes	NS	Yes	No	9 (Fair)
Stewart R.E., 1991	Ex. SPECT	Yes	Yes	Yes	Yes	No	Yes	Yes	Yes	Yes	No	Yes	Yes	Yes	No	11 (Good)
Salustri A. et al. 1992	EST, Echo dobu	Yes	Yes	Yes	Yes	No	Yes	Yes	No	Yes	No	Yes	Yes	Yes	No	10 (Fair)
Martin T.W., 1992	Echo dobu, Echo dipy	Yes	Yes	Yes	Yes	No	Yes	Yes	No	Yes	No	Yes	Yes	Yes	No	10 (Fair)
Quiñones M.A. et al. 1992	ESE, ex. SPECT	Yes	Yes	Yes	Yes	No	Yes	Yes	No	Yes	No	Yes	Yes	Yes	No	10 (Fair)
Prisant L.M., 1992	Ex. SPECT	Yes	Yes	Yes	Yes	No	Yes	Yes	Yes	Yes	No	Yes	Yes	Yes	No	11 (Good)
Gupta N.C., 1992	Ex. SPECT, adeno SPECT	Yes	Yes	Yes	Yes	No	Yes	Yes	Yes	Yes	No	Yes	Yes	Yes	No	11 (Good)
Marwick T., 1993	Echo dobu, dobu SPECT	Yes	Yes	Yes	Yes	No	Yes	Yes	Yes	Yes	No	Yes	Yes	Yes	No	11 (Good)
Takeuchi M., 1993	Echo dobu, dipy SPECT	Yes	Yes	Yes	Yes	No	Yes	Yes	Yes	Yes	No	Yes	Yes	Yes	No	11 (Good)
van Rugge F.P. et al. 1993	EST, DobuCMR	Yes	Yes	Yes	Yes	No	Yes	Yes	No	Yes	No	Yes	Yes	Yes	No	10 (Fair)
Hecht H.S. et al. 1993	ESE, ex-SPECT	Yes	Yes	Yes	Yes	No	Yes	Yes	Yes	Yes	No	Yes	Yes	Yes	Yes	12 (Good)
Cohen J.L. et al. 1993	ESE, echo dobu	Yes	Yes	Yes	Yes	No	Yes	Yes	Yes	Yes	No	Yes	Yes	Yes	No	11 (Good)
Baer F.M., 1993	DipyCMR	Yes	Yes	Yes	Yes	No	Yes	Yes	Yes	Yes	No	Yes	Yes	Yes	No	11 (Good)
Beleslin B.D., 1994	ESE, Echo dobu, echo dipy	Yes	Yes	Yes	Yes	No	Yes	Yes	Yes	Yes	No	Yes	Yes	Yes	No	11 (Good)
Panza J.A. et al. 1994	EST, echo dobu	Yes	Yes	Yes	Yes	No	Yes	Yes	No	Yes	No	Yes	Yes	Yes	No	10 (Fair)
Zammarchi A., 1994	Ex. SPECT	Yes	Yes	Yes	Yes	No	Yes	Yes	Yes	Yes	No	Yes	NS	Yes	No	10 (Fair)
Hartnell G., 1994	DipyCMR	Yes	Yes	Yes	Yes	No	Yes	Yes	Yes	Yes	No	Yes	Yes	Yes	No	11 (Good)
Dagianti A. et al. 1995	ESE, echo dobu, echo dipy	Yes	Yes	Yes	Yes	No	Yes	Yes	Yes	Yes	No	Yes	Yes	Yes	No	11 (Good)

Ho F.M., 1995	Echo dobu, dipy SPECT	Yes	Yes	Yes	Yes	No	Yes	Yes	Yes	Yes	No	Yes	Yes	Yes	No	11 (Good)
Bjørnstad K. et al. 1995	ESE, echo dipy	Yes	Yes	Yes	Yes	No	Yes	Yes	Yes	Yes	No	Yes	Yes	Yes	No	11 (Good)
Fleming R.M., 1995	Ex. SPECT, dobu SPECT, dipy SPECT	Yes	Yes	Yes	Yes	No	Yes	Yes	No	Yes	No	Yes	NS	Yes	No	9 (Fair)
San Román J.A. et al. 1996	EST, echo dobu, echo dipy	Yes	Yes	Yes	Yes	No	Yes	Yes	No	Yes	No	Yes	Yes	Yes	No	10 (Fair)
Kisacik H.L. et al. 1996	EST, echo dobu, dobu SPECT	Yes	Yes	Yes	Yes	No	Yes	Yes	Yes	Yes	No	Yes	Yes	Yes	No	11 (Good)
Pingitore A., 1996	Echo dobu, echo dipy	Yes	Yes	Yes	Yes	No	Yes	Yes	Yes	Yes	No	Yes	Yes	Yes	No	11 (Good)
Cramer M.J., 1996	Dipy SPECT, adeno SPECT	Yes	Yes	Yes	Yes	No	Yes	Yes	No	Yes	No	Yes	Yes	Yes	No	10 (Fair)
Laurienzo J.M. et al. 1997	EST	Yes	Yes	Yes	Yes	No	Yes	Yes	No	Yes	No	Yes	Yes	Yes	No	10 (Fair)
Morise A.P. et al. 1997	EST	Yes	Yes	Yes	Yes	No	Yes	Yes	Yes	Yes	No	Yes	Yes	Yes	No	11 (Good)
Hennessy T.G. et al. 1997	EST, echo dobu	Yes	Yes	Yes	Yes	No	Yes	Yes	Yes	Yes	No	Yes	NS	Yes	No	10 (Fair)
Zhao S., 1997	DipyCMR	Yes	Yes	Yes	Yes	No	Yes	Yes	Yes	Yes	No	Yes	Yes	Yes	No	11 (Good)
Ho Y.L. et al. 1998	EST	Yes	Yes	Yes	Yes	No	Yes	Yes	Yes	Yes	No	Yes	Yes	Yes	No	11 (Good)
Kalaria V.G. et al. 1998	EST	Yes	Yes	Yes	Yes	No	Yes	Yes	Yes	Yes	No	Yes	Yes	Yes	No	11 (Good)
Santoro G.M. et al. 1998	EST, echo dobu, echo dipy, dobu SPECT, dipy SPECT	Yes	Yes	Yes	Yes	No	Yes	Yes	Yes	Yes	No	Yes	Yes	Yes	No	11 (Good)
San Román J.A. et al. 1998	EST, echo dobu, echo dipy, dobu SPECT	Yes	Yes	Yes	Yes	No	Yes	Yes	Yes	Yes	No	Yes	Yes	Yes	No	11 (Good)
Elhendy A., 1998	Echo dobu, dobu SPECT	Yes	Yes	Yes	Yes	No	Yes	Yes	Yes	Yes	No	Yes	Yes	Yes	No	11 (Good)
Badruddin S.M. et al. 1999	ESE	Yes	Yes	Yes	Yes	No	Yes	Yes	No	Yes	No	Yes	Yes	Yes	No	10 (Fair)

Fragasso G., 1999	Echo dobu, echo dipy, ex. SPECT	Yes	Yes	Yes	Yes	No	Yes	Yes	Yes	Yes	No	Yes	Yes	Yes	Yes	12 (Good)
Previtali M., 1999	Echo dobu, ex. SPECT	Yes	Yes	Yes	Yes	No	Yes	Yes	Yes	Yes	No	Yes	Yes	Yes	No	11 (Good)
Nagel E., 1999	DobuCMR	Yes	Yes	Yes	Yes	No	Yes	Yes	Yes	Yes	No	Yes	Yes	Yes	No	11 (Good)
Ciaroni S., 2000	Echo dobu, ex. SPECT	Yes	Yes	Yes	Yes	No	Yes	Yes	No	Yes	No	Yes	NS	Yes	No	9 (Fair)
Smart S.C., 2000	Echo dobu	Yes	Yes	Yes	Yes	No	Yes	Yes	Yes	Yes	No	Yes	Yes	Yes	No	11 (Good)
Geleijnse M.L., 2000	Echo dobu	Yes	Yes	Yes	Yes	No	Yes	Yes	Yes	Yes	No	Yes	Yes	Yes	No	11 (Good)
Elhendy A., 2000	Echo dobu, dobu SPECT	Yes	Yes	Yes	Yes	No	Yes	Yes	Yes	Yes	No	Yes	Yes	Yes	No	11 (Good)
Doğruca Z., 2000	Ex. SPECT	Yes	Yes	Yes	Yes	No	Yes	Yes	Yes	Yes	No	Yes	NS	Yes	No	10 (Fair)
Smart S.C., 2000	Dipy SPECT	Yes	Yes	Yes	Yes	No	Yes	Yes	Yes	Yes	No	Yes	Yes	Yes	No	11 (Good)
Gentile R. et al. 2001	EST, ex. SPECT	Yes	Yes	Yes	Yes	No	Yes	Yes	Yes	Yes	No	Yes	Yes	Yes	No	11 (Good)
Tandoğan I., 2001	Echo dobu, ex. SPECT	Yes	Yes	Yes	Yes	No	Yes	Yes	Yes	Yes	No	Yes	Yes	Yes	No	11 (Good)
Lancellotti P., 2001	Echo dobu, dobu SPECT	Yes	Yes	Yes	Yes	No	Yes	Yes	Yes	Yes	No	Yes	Yes	Yes	No	11 (Good)
Vigna C. et al. 2001	Echo dipy	Yes	Yes	Yes	Yes	No	Yes	Yes	No	Yes	No	Yes	Yes	Yes	No	10 (Fair)
Bokhari S. et al. 2002	EST	Yes	Yes	Yes	Yes	No	Yes	Yes	Yes	Yes	No	Yes	Yes	Yes	No	11 (Good)
Rollán M.J. et al. 2002	EST	Yes	Yes	Yes	Yes	No	Yes	Yes	No	Yes	No	Yes	Yes	Yes	No	10 (Fair)
Ha J.W. et al. 2002	ESE	Yes	Yes	Yes	Yes	No	Yes	Yes	Yes	Yes	No	Yes	Yes	Yes	No	11 (Good)
al-Saadi N., 2002	DobuCMR	Yes	Yes	Yes	Yes	No	Yes	Yes	No	Yes	No	Yes	Yes	Yes	No	10 (Fair)
Cortigiani L. et al. 2003	EST, echo dipy	Yes	Yes	Yes	Yes	No	Yes	Yes	Yes	Yes	No	Yes	Yes	Yes	No	11 (Good)
Shin J.H. et al. 2003	ESE	Yes	Yes	Yes	Yes	No	Yes	Yes	Yes	Yes	No	Yes	Yes	Yes	Yes	12 (Good)
Rigo F. et al. 2003	Dual imag.	Yes	Yes	Yes	Yes	No	Yes	Yes	Yes	Yes	No	Yes	Yes	Yes	No	11 (Good)
Lowenstein J. et al. 2003	Dual imag.	Yes	Yes	Yes	Yes	No	Yes	Yes	No	Yes	No	Yes	NS	Yes	No	9 (Fair)
Nohtomi Y. et al. 2003	Dual imag.	Yes	Yes	Yes	Yes	No	Yes	Yes	Yes	Yes	No	Yes	Yes	Yes	No	11 (Good)
Olszowska M., 2003	Dobu SPECT	Yes	Yes	Yes	Yes	No	Yes	Yes	Yes	Yes	No	Yes	Yes	Yes	No	11 (Good)
Rerkpattanapipat P., 2003	Ex. CMR	Yes	Yes	Yes	No	No	Yes	Yes	No	Yes	No	Yes	Yes	Yes	No	9 (Fair)
Zeng H. et al. 2004	EST	Yes	Yes	Yes	Yes	No	Yes	Yes	Yes	Yes	No	Yes	NS	Yes	No	10 (Fair)
Senior R., 2004	Dipy SPECT	Yes	Yes	Yes	Yes	Yes	Yes	Yes	Yes	Yes	No	Yes	Yes	Yes	No	12 (Good)
Wahl A., 2004	DobuCMR	Yes	Yes	Yes	Yes	No	Yes	Yes	Yes	Yes	No	Yes	Yes	Yes	No	11 (Good)
Takase B., 2004	DipyCMR	Yes	Yes	Yes	Yes	No	Yes	Yes	Yes	Yes	No	Yes	Yes	Yes	No	11 (Good)
Paetsch I., 2004	DobuCMR, adenoCMR	Yes	Yes	Yes	Yes	No	Yes	Yes	Yes	Yes	No	Yes	Yes	Yes	No	11 (Good)

González P. et al. 2005	EST, ex. SPECT	Yes	Yes	Yes	Yes	No	Yes	Yes	No	Yes	No	Yes	NS	Yes	No	9 (Fair)
Nedeljkovic I. et al. 2006	EST, echo dobu, echo dipy	Yes	Yes	Yes	Yes	No	Yes	Yes	Yes	Yes	No	Yes	Yes	Yes	No	11 (Good)
Ascione L. et al. 2006	Dual imag.	Yes	Yes	Yes	Yes	No	Yes	Yes	Yes	Yes	No	Yes	Yes	Yes	No	11 (Good)
Jeetley P., 2006	Dipy SPECT	Yes	Yes	Yes	Yes	No	Yes	Yes	Yes	Yes	No	Yes	Yes	Yes	No	11 (Good)
Karavidas A.I., 2006	AdenoSPECT	Yes	Yes	Yes	Yes	No	Yes	Yes	No	Yes	No	Yes	Yes	Yes	Yes	11 (Good)
Vigna C. et al. 2006	Echo dipy, dipy SPECT	Yes	Yes	Yes	Yes	No	Yes	Yes	No	Yes	No	Yes	Yes	Yes	No	10 (Fair)
Klem I., 2006	AdenoCMR	Yes	Yes	Yes	Yes	No	Yes	Yes	Yes	Yes	No	Yes	Yes	Yes	Yes	12 (Good)
Michaelides A.P. et al. 2007	EST	Yes	Yes	Yes	Yes	No	Yes	Yes	No	Yes	No	Yes	Yes	Yes	No	10 (Fair)
Aggeli C., 2007	AdenoSPECT	Yes	Yes	Yes	Yes	No	Yes	Yes	Yes	Yes	No	Yes	Yes	Yes	No	11 (Good)
Stauder N.I., 2007	DipyCMR	Yes	Yes	Yes	Yes	No	Yes	Yes	Yes	Yes	No	Yes	Yes	Yes	No	11 (Good)
Bokhari S. et al. 2008	EST, ex. SPECT	Yes	Yes	Yes	Yes	No	Yes	Yes	Yes	Yes	No	Yes	Yes	Yes	No	11 (Good)
Müller H. et al. 2008	ESE	Yes	Yes	Yes	Yes	No	Yes	Yes	No	Yes	No	Yes	Yes	Yes	No	10 (Fair)
Futamatsu H., 2008	AdenoSPECT, adeno CMR	Yes	Yes	Yes	Yes	No	Yes	Yes	Yes	Yes	No	Yes	Yes	Yes	No	11 (Good)
Kelle S., 2008	DobuCMR	Yes	Yes	Yes	Yes	No	Yes	Yes	No	Yes	No	Yes	Yes	Yes	No	10 (Fair)
Klein C., 2008	AdenoCMR	Yes	Yes	Yes	Yes	No	Yes	Yes	Yes	Yes	No	Yes	Yes	Yes	No	11 (Good)
Pingitore A., 2008	DipyCMR	Yes	Yes	Yes	Yes	No	Yes	Yes	Yes	Yes	No	Yes	Yes	Yes	Yes	12 (Good)
Heilmaier C., 2009	DobuCMR	Yes	Yes	Yes	Yes	No	Yes	Yes	Yes	Yes	No	Yes	Yes	Yes	No	11 (Good)
Lu C. et al. 2010	EST	Yes	Yes	Yes	Yes	No	Yes	Yes	Yes	Yes	No	Yes	Yes	Yes	No	11 (Good)
Gaibazzi N. et al. 2010	Dual imag.	Yes	Yes	Yes	Yes	No	Yes	Yes	Yes	Yes	No	Yes	Yes	Yes	No	11 (Good)
Gebker R., 2010	DobuCMR	Yes	Yes	Yes	Yes	No	Yes	Yes	Yes	Yes	No	Yes	Yes	Yes	No	11 (Good)
Arnold J.R., 2010	AdenoCMR	Yes	Yes	Yes	Yes	No	Yes	Yes	Yes	Yes	No	Yes	Yes	Yes	No	11 (Good)
Cortigiani L. et al. 2011	Dual imag.	Yes	Yes	Yes	Yes	No	Yes	Yes	Yes	Yes	Yes	Yes	Yes	Yes	Yes	13 (Good)
Greulich S. et al. 2012	EST	Yes	Yes	Yes	Yes	No	Yes	Yes	Yes	Yes	No	Yes	Yes	Yes	No	11 (Good)
Weustink A.C. et al. 2012	EST, ex. SPECT	Yes	Yes	Yes	Yes	No	Yes	Yes	No	Yes	No	Yes	Yes	Yes	No	10 (Fair)
Kasprzak J.D. et al. 2013	Dual imag.	Yes	Yes	Yes	Yes	No	Yes	Yes	Yes	Yes	No	Yes	Yes	Yes	No	11 (Good)
Bettencourt N., 2013	AdenoCMR	Yes	Yes	Yes	Yes	No	Yes	Yes	Yes	Yes	No	Yes	Yes	Yes	No	11 (Good)
Greenwood .JP., 2014	AdenoSPECT, adeno CMR	Yes	Yes	Yes	Yes	No	Yes	Yes	Yes	Yes	No	Yes	Yes	Yes	No	11 (Good)
Mordi I., 2014	DobuCMR	Yes	Yes	Yes	Yes	No	Yes	Yes	Yes	Yes	No	Yes	Yes	Yes	No	11 (Good)
Mordini F.E., 2014	DipyCMR	Yes	Yes	Yes	Yes	No	Yes	Yes	Yes	Yes	No	Yes	Yes	Yes	No	11 (Good)
Deva D.P., 2014	DipyCMR	Yes	Yes	Yes	Yes	No	Yes	Yes	No	Yes	No	Yes	NS	Yes	No	9 (Fair)
Salerno M., 2014	AdenoCMR	Yes	Yes	Yes	Yes	No	Yes	Yes	Yes	Yes	No	Yes	Yes	Yes	No	11 (Good)

Yun C.H., 2015	DipyCMR	Yes	Yes	Yes	Yes	No	Yes	Yes	Yes	Yes	No	Yes	Yes	Yes	No	11 (Good)
Manka R., 2015	AdenoCMR	Yes	Yes	Yes	Yes	No	Yes	Yes	Yes	Yes	No	Yes	Yes	Yes	No	11 (Good)
Raman S.V., 2016	Ex. SPECT, ex. CMR	Yes	Yes	Yes	Yes	No	Yes	Yes	Yes	Yes	Yes	Yes	Yes	Yes	No	12 (Good)
Ahmad I.G., 2016	Ex. SPECT, adeno CMR	Yes	Yes	Yes	Yes	No	Yes	Yes	Yes	Yes	No	Yes	Yes	Yes	No	11 (Good)
Attar A. et al. 2017	EST	Yes	Yes	Yes	Yes	No	Yes	Yes	No	Yes	No	Yes	NS	Yes	No	9 (Fair)
Ntsinjana H.N., 2017	AdenoCMR	Yes	Yes	Yes	Yes	No	Yes	Yes	No	Yes	No	Yes	Yes	Yes	No	10 (Fair)
Foley J.R.J., 2017	AdenoCMR	Yes	Yes	Yes	Yes	No	Yes	Yes	Yes	Yes	No	Yes	Yes	Yes	No	11 (Good)
Pichel I.Á. et al. 2019	Dual imag.	Yes	Yes	Yes	Yes	No	Yes	Yes	Yes	Yes	No	Yes	Yes	Yes	No	11 (Good)
Weberling L.D., 2023	DobuCMR	Yes	Yes	Yes	Yes	No	Yes	Yes	Yes	Yes	No	Yes	NS	Yes	No	10 (Fair)
Ochs A., 2025	Ex. CMR	Yes	Yes	Yes	Yes	No	Yes	Yes	Yes	Yes	No	Yes	Yes	Yes	No	11 (Good)

Table S2. Subgroup analysis by region, stratified by modality

Modality	Region	N studies	Total sample	Sensitivity (95% CI)	Specificity (95% CI)
EST	Asia	3	1029	91.7% (77.3–98.3%)	27.0% (11.9–34.4%)
EST	Europe	15	1599	69.7% (66.6–76.9%)	61.5% (50.3–65.1%)
EST	North America	6	2181	42.1% (26.6–53.9%)	66.3% (57.6–75.9%)
EST	South America	1	145	37.0% (37.0–37.0%)	74.0% (74.0–74.0%)
SE	Asia	3	284	90.0% (85.0–94.0%)	78.4% (65.0–93.0%)
SE	Europe	40	4515	79.0% (79.2–83.4%)	85.7% (80.9–88.3%)
SE	North America	11	1892	83.8% (82.7–85.4%)	83.9% (80.2–86.6%)
SE	Other	2	2089	87.6% (87.0–89.0%)	77.3% (76.0–80.0%)
SE	South America	1	752	86.8% (86.8–86.8%)	73.2% (73.2–73.2%)
SPECT	Asia	2	174	91.8% (89.0–98.0%)	81.3% (73.0–85.0%)

SPECT	Europe	25	2609	79.5% (73.2–81.6%)	72.3% (73.0–79.4%)
SPECT	North America	15	1818	85.6% (81.7–90.6%)	77.7% (70.7–84.5%)
SPECT	South America	1	145	87.0% (87.0–87.0%)	57.0% (57.0–57.0%)
CMR	Asia	2	160	87.2% (77.0–93.0%)	83.2% (80.0–85.0%)
CMR	Europe	23	3153	81.3% (79.8–85.7%)	87.6% (84.0–86.6%)
CMR	North America	9	467	83.1% (80.8–85.3%)	85.9% (71.5–95.5%)

Table S3. Subgroup analysis by study period, stratified by modality

Modality	Period	N studies	Total sample	Sensitivity (95% CI)	Specificity (95% CI)
EST	1990–2004	17	3120	52.4% (34.5–53.4%)	65.5% (60.1–71.6%)
EST	2005–2025	8	1834	76.0% (71.2–95.7%)	42.0% (15.8–46.5%)
SE	1990–2004	47	6381	81.1% (82.2–84.9%)	84.3% (79.0–84.6%)
SE	2005–2025	10	3151	86.2% (86.4–87.8%)	78.2% (75.6–78.3%)

SPECT	1990–2004	32	2929	85.6% (82.2–89.4%)	72.5% (67.4–78.9%)
SPECT	2005–2025	11	1817	77.6% (72.0–82.7%)	77.0% (75.3–81.2%)
CMR	1990–2004	11	794	82.3% (76.6–88.7%)	86.3% (84.1–88.0%)
CMR	2005–2025	23	2986	81.6% (79.6–85.6%)	87.4% (83.7–86.7%)

Table S4. Subgroup analysis by study design, stratified by modality

Modality	Design	N studies	Total sample	Sensitivity (95% CI)	Specificity (95% CI)
EST	Prospective	18	2511	61.6% (54.2–61.1%)	63.3% (57.4–62.3%)
EST	Retrospective	7	2443	60.8% (31.0–90.6%)	50.1% (21.8–72.8%)
SE	Prospective	55	8520	82.7% (85.1–86.6%)	82.3% (77.0–80.1%)
SE	Retrospective	2	1012	83.8% (82.0–86.0%)	82.1% (78.0–87.0%)
SPECT	Prospective	40	4020	81.6% (74.8–82.1%)	74.4% (73.5–80.2%)
SPECT	Retrospective	3	726	87.4% (83.8–90.9%)	73.4% (65.4–79.0%)

CMR	Prospective	33	3761	81.8% (80.3–85.5%)	87.1% (83.9–86.4%)
CMR	Retrospective	1	19	82.0% (82.0–82.0%)	100.0% (100.0–100.0%)
